# Albumin-Keratin Casts Obstruct Renal Tubular and Vascular Lumens Following Kidney Ischemia

**DOI:** 10.1016/j.ekir.2026.106356

**Published:** 2026-02-12

**Authors:** Martina Bryant, Olivia Boykin, Olivia Mosley, Angela Ajith, Oliver Oglesby, Konstantin Kurzin, Carlee Harris, Sadaf Husan, Daniela Dokam, Sarah R. McLarnon, Jingping Sun, Brendan Marshall, Amanda Barrett, Aaron Polichnowski, Wendy B. Bollag, David Mattson, Paul M. O’Connor

**Affiliations:** 1 Department of Physiology, Medical College of Georgia, Augusta University, Augusta, Georgia, USA; 2 Department of Cell Biology and Physiology, School of Medicine, University of North Carolina, Chapel Hill, North Carolina, USA; 3 Department of Anatomy and Cell Biology, Medical College of Georgia, Augusta University, Augusta, Georgia, USA; 4 Department of Pathology, Medical College of Georgia, Augusta University, Augusta, Georgia, USA; 5 Department of Biomedical Sciences, Quillen College of Medicine, East Tennessee State University, Johnson City, Tennessee, USA

**Keywords:** acute kidney injury, casts, cytokeratin, ischemia-reperfusion, red blood cell trapping

## Abstract

**Introduction:**

We have recently reported that in ischemic acute kidney injury (AKI), red blood cell (RBC) trapping causes fluid extravasation from the congested capillary circulation and subsequent toxic injury to the surrounding tubules. The goal of this study was to identify the cause of the obstruction leading to RBC trapping.

**Methods:**

Studies were performed in Sprague Dawley rats and human kidneys. Rats underwent warm arterial clamp ischemia for 45 minutes with 0 to 24 hours of reperfusion. New blood entering the kidney upon reperfusion of the kidney was tracked using Evans blue dye. At harvest, kidneys were fixed and histological analysis performed. Vascular and tubular casts from 4 human and 3 rat kidneys were isolated using laser dissection microscopy and their contents determined by mass spectrometry shotgun proteomics.

**Results:**

New blood entering the kidney appeared to be impeded in large renal veins by a homogeneous substance which stained blue in trichrome sections. Scanning electron microscopy revealed the substance to be a solid that conformed the shape of the encased RBCs. A similar substance was observed in the lumens of many tubules and small capillaries. Proteomic analysis identified albumin and keratins as major proteins present in the casts, and this was confirmed using immunohistochemistry.

**Conclusion:**

Our data indicate that albumin-keratin casts obstruct both vascular and tubular structures in the kidney following a period of ischemia. These casts may be the primary cause of vascular obstruction post-ischemia and likely lead to RBC trapping and associated acute tubular injury.

## Graphical abstract

**Figure:**
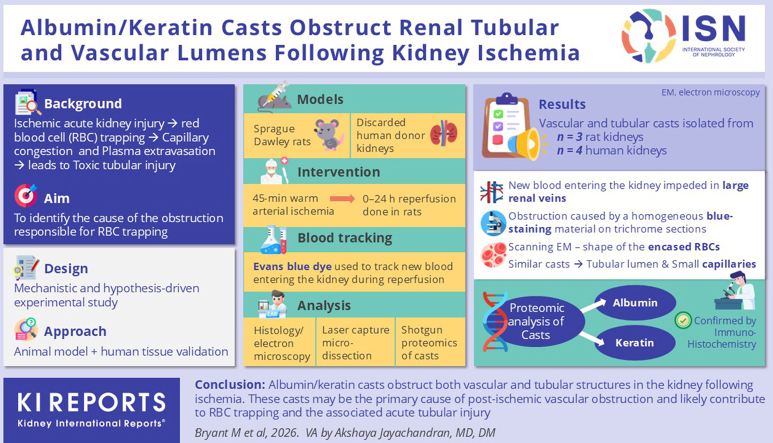

Following ischemic AKI, the kidneys appear swollen and pale, with a dark red outer medulla (RBC trapping).^1^, ^2^, ^3^, ^4^, ^5^, ^6^, ^7^, ^8^, ^9^ RBC trapping, which presents as densely packed RBCs within the renal outer medullary circulation,^4^^,^^10^ is associated with severe tubular injury in both humans^7^^,^^11^ and in animal models^10^^,^^12^; and maneuvers to reduce RBC trapping have been shown to improve kidney function after ischemia in rats.^12^^,^^13^ We have recently reported that RBC trapping promotes toxic tubular injury, secondary to the extravasation of blood proteins from the congested renal vasculature, which are then taken up by nearby tubular cells.^14^ Unfortunately, current approaches to limit RBC trapping are not easily translated to the clinic, and the development of new approaches to prevent RBC trapping are hindered by a lack of understanding of the pathophysiological processes responsible for this phenomenon.

Considering that RBCs accumulate in the medullary circulation upon reperfusion of the kidney,^15^^,^^16^ this suggests that blood can enter the renal medullary circulation, but it cannot exit. The goal of the current study was to localize the site of obstruction responsible for RBC trapping following a period of warm ischemia. In doing this, we identified a solid substance within the renal venous circulation which appeared to be responsible for obstructing blood flow. Given this, a secondary goal of this study became to identify the nature of this substance.

## Methods

### Animals

All experiments were conducted in accordance with the National Institutes of Health “Guide for the Care and Use of Laboratory Animals” and were approved and monitored by the Augusta University (AU) Institutional Animal Care and Use Committee. Age-matched male and female Sprague Dawley rats were used in all experiments. Rats were housed in temperature-controlled (20–26 °C) and humidity-controlled (30%–70%), 12:12-hour light-cycled conventional animal quarters. The rats were provided *ad libitum* access to water and standard 18%-protein rodent chow (Envigo Teklad, 2918).

### Warm Bilateral Ischemia-Reperfusion Surgery

Ischemia-reperfusion was performed as previously described.^15^^,^^17^^,^^18^ Briefly, animals were anesthetized with approximately 3% isoflurane and 95% oxygen. Body temperature (rectal probe) was maintained at approximately 37 °C by servo-controlled heating table and infrared heat lamp (R40, Satco S4998). The renal pedicles were accessed via flank incisions and the renal arteries isolated from the renal veins to allow placement of arterial clamps. Depending on the experimental protocol, either 1 or both renal arteries were clamped with Schwartz Micro Serrefines (Fine Science Tools #18052-03, Foster City, CA) followed by reperfusion. In some animals in which tracking of new blood perfusion into the kidney was performed, the left kidney was placed in a kidney cup. The kidneys were fixed with VIP-Fixative (Fisher #23-730-587) or electron microscopy fixative (4% paraformaldehyde and 2% glutaraldehyde in 0.1 M sodium cacodylate buffer, pH: 7.4) for later histological analysis. Animals that recovered from anesthesia were given buprenorphine for analgesia.

### Quantifying Plasma Extravasation

Evans blue binds strongly to plasma proteins creating a large molecular weight tracer, which can be used to investigate plasma extravasation in tissues.^19^, ^20^, ^21^ To confirm the extravasation of blood proteins, we injected 50 mg/kg of Evans blue in 1 mL of saline into the tail vein of rats approximately 5 minutes before removal of the arterial clamp. Kidneys were then allowed to reperfuse for 0, 1, or 3 hours before being harvested and weighed and placed in fixative solution.

### Tracking the Path of New Blood Entering the Kidney

Evans blue was used to distinguish between new blood that had entered the kidney during reperfusion from blood that was present in the kidney vasculature during ischemia. After injecting Evans blue, RBC’s that entered the kidney after reperfusion could then be distinguished from RBC’s that were already present by their blue coloring.

### Human Kidney Tissue

Human kidneys were obtained from LifeLink Foundation, which provides human donor organs and tissues (nontransplantable) for research purposes and the National Institutes of Health National Disease Research Interchange. LifeLink is an approved Organ Procurement Organization in Georgia with a primary mission of recovering donor organs and tissues for human transplantation. Upon authorization for research, human kidneys deemed unsuitable for transplant together with deidentified clinical and medical history of the donor were made available to AU, a LifeLink-approved research entity that has agreed to receive such donor organs and tissues for use in studies under AU Institutional Review Board 1732545. Kidney procurement through the National Institutes of Health National Disease Research Interchange was approved by AU Institutional Review Board 2288349-1. Reasons for kidney discard were not always provided; however, known reasons included poor pump perfusion, surgical damage on harvest, unable to find recipient match, and significant glomerular sclerosis on biopsy.

### Scanning Electron Microscopy

Tissue was fixed overnight before dehydration with a graded ethanol series (25%–100%, 10–15 minutes each). Dehydrated tissue was then critical point dried from liquid CO_2_ in a Leica EM CPD300 critical point drier (Leica Microsystems Inc., Deerfield, IL). Following critical point drying, tissue was mounted on aluminum stubs with carbon adhesive tabs and sputter-coated for 10 minutes with gold-palladium in a Leica EM ACE200 sputter coater (Leica Microsystems Inc., Deerfield, IL), followed by observation and imaging in a JSM IT-500HR scanning electron microscope (JEOL, Peabody, MA) running at 15kV.

### Laser Dissection Microscopy and Mass spectrometry

Paraffin-embedded tissue blocks were sectioned at 5-μm thickness using a rotary microtome. Sections were mounted onto Leica PEN-Membrane slides (Cat. No. 11600288) and air-dried at room temperature before staining. The slides were then deparaffinized and stained with hematoxylin and eosin. Stained slides were air-dried completely at room temperature for 24 hours without xylene clearing or cover slipping to preserve membrane integrity and compatibility with laser capture. The slides were loaded into the Leica LMD6 system. Regions of interest were visualized and micro-dissected using the integrated laser guidance software. Dissected material was collected into protein-compatible dry collection tube caps, for downstream mass spectrometry protein analysis by the AU Proteomics and Mass Spectrometry Core Laboratory following a previously published protocol.^22^ The estimated percentage of cast material made up of a specific protein was calculated from peptide spectrum match with percentage of cast material = average peptide spectrum match–specific protein / total peptide spectrum match of all proteins identified in sample.^23^^,^^24^

For the renal venous cast sample from rats, tissue was dissected and collected from predominantly cell-free areas of interest within large venous casts from 2 rats, until approximately 2.5 to 5 mm^2^ was collected (Supplementary Figure S1A–C). Three individual venous cast samples from these 2 rats were then analyzed separately using mass spectrometry, and only the proteins that appeared in all 3 samples and contributed > 1% of total cast protein by peptide spectrum match were reported as being present in the casts. A similar sized control sample from tissue outside the cast region was taken. In another rat sample, approximately 2.5 to 5 mm^2^ of sample was collected from regions of interest containing outer medullary vascular bundles filled with cast material. Tubular cast samples were collected from 4 individual human samples and were not pooled. Two samples were collected from large tubular casts from dedifferentiated tubules around the cortical medullary border (Supplementary Figure S1D–F). Another 2 samples were taken from large casts in the papilla. Approximately 2.5 to 5 mm^2^ of sample was collected along for each sample.

### Immunostaining

Immunostaining was performed as previously described.^15^ All sections were incubated with primary antibody in 10% goat serum in 0.1% phosphate-buffered saline with Tween overnight at 4 °C. For albumin staining, albumin recombinant rabbit monoclonal antibody, (Invitrogen cat# MA5-32531) was used at 1:1000 dilution. For Pan-keratin staining, the slides were incubated with anti-keratin rabbit polyclonal antibody (ab9377 Abcam) at 1:200. For keratin-10, rabbit polyclonal antibody (18343-1-AP Proteintech) was used at 1:1000. For keratin-6A, rabbit polyclonal antibody (10590-1-AP Proteintech) was used at 1:400. For keratin-1, a purified rabbit polyclonal antibody (905601 BioLegend) was used at 1:400. For keratin-5, a purified rabbit polyclonal antibody (905504 BioLegend) was used at 1:500. The next day, the slides were washed 3 times in 0.1% phosphate-buffered saline with Tween for 5 minutes each time before being incubated with a secondary antibody (goat anti-rabbit IgG-HRP [ab6721 Abcam]) at 1:400 dilution in 10% goat serum in 0.1% phosphate-buffered saline with Tween for 50 minutes at room temperature. The slides were then washed 3 times in 0.1% phosphate-buffered saline with Tween for 5 minutes each time before chromogen-staining with 3,3'-Diaminobenzidine (DAB) for 5 minutes. Slides were then rinsed 3 times in distilled water before being counterstained with hematoxylin for 1 minute and washed in running tap water. Slides were covered using Cytoseal XYL medium (Thermo Scientific cat#8312-4). No primary antibody was used as a control.

### Endogenous DAB Staining

DAB staining was used to visualize the endogenous peroxidase activity of hemoglobin.^14^^,^^25^ Rehydrated sections were incubated with DAB solution (Biocare Medical Betazoid DAB Kit cat# BDB2004L) for 20 minutes, followed by rinsing in distilled water. Stained sections were counterstained with hematoxylin for 1 minute before being dehydrated in an ethanol series and cleared in xylene for mounting.

### Heparin Studies

Ischemia-reperfusion was performed as described above. Thirty minutes before placing the arterial clamp, either 1 ml of saline (vehicle) or high dose heparin (300 IU/kg) in 1 ml of saline was injected into the tail vein. After 45 minutes of warm arterial clamp ischemia, the clamps were removed, and the kidneys were allowed to reperfuse for 1 hour before being harvested and fixed for analysis. Scanning electron microscopy analysis of the large renal veins was then performed on 4 heparin (2 males/2 females) and 4 (2 males/2 females) control rats to identify whether casts were present.

### Semiquantitative Scoring

Whole slides containing human kidney tissue were examined by an investigator blinded to the source of the samples. Keratin positive tubular casts were scored on a 0 to 3 scale for both the cortex and medulla, with 0 to 1 representing a minimal number of casts, 2 representing a moderate number of casts, and 3 indicating casts were common. Acute tubular injury consisting of tubular cell sloughing, flattening and dilation of tubules, and necrotic tubular cells was scored at 20 × magnification with scores of 0 to 3, with 0 to 1 representing minimal acute tubular injury, 2 representing moderate tubular injury (some injured tubules in each field), and 3 representing severe tubular injury (many injured tubules in each field).

### Automated Image Quantification

Trichrome-stained whole slides were scanned and digitized using a slide scanner (Zeiss Axioscan 7, Zeiss, USA). Red-blue cast quantification was performed on digitized whole kidney trichrome sections using open-source software for digital pathology image analysis (QuPath)^26^ trained to identify red-blue–colored casts according to a previously published method.^27^

### Statistics

Graphpad Prizm was used for all data analysis. Animal treatment was randomized for all groups studied and groups alternated to avoid time-of-cage effects. Histological scoring was either automated or performed by an investigator unaware of the source of the samples. No data were excluded for any reason other than a failed surgery before analysis.

## Results

**Figure 1:**
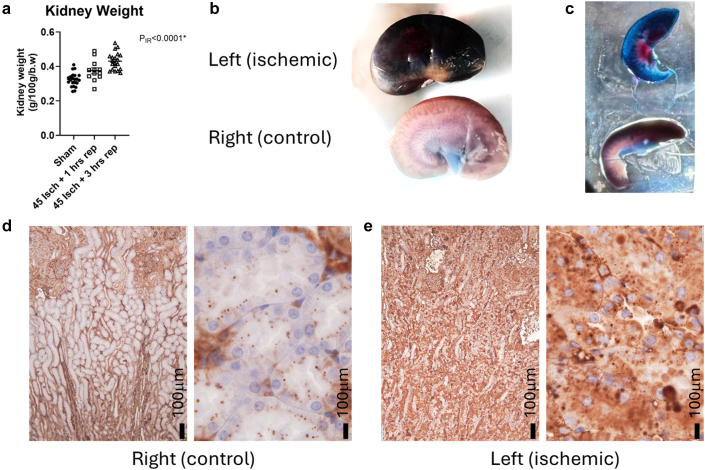
Forty-five minutes of warm arterial clamping causes renal venous obstruction resulting in rapid extravasation of plasma upon reflow. (a) Wet kidney weights per 100 g or body weight in sham surgery time control rats without arterial clamping (closed circles, *n* = 24 kidneys, from 10 females and 5 males), rats following 45 minutes of arterial clamping with 1 hour of reperfusion (open squares, *n* = 12 kidneys, 10 females and 2 ales) and rats following 45 minutes of arterial clamping with 3 hours of reperfusion (closed triangles, *n* = 20 kidneys, 10 males). Data are mean ± SE. *P*_IR_ is the result of 1-way analysis of variance comparing kidney weights. (b and c) Representative images of bisected kidneys from a single rat treated with Evans blue in which the left kidney (ischemic) underwent 45 minutes of ischemia with 1 hour of reperfusion and the right kidney (control) was left unclamped. (c) Consistent with the extravasation of Evans blue–labelled blood proteins from the vasculature, in the ischemic kidney, the entire kidney parenchyma has turned dark blue. In the control kidney, only the blood vessels appear blue against the pale background of the cortical parenchyma. (d) In control kidneys in the boundary zone of the cortex and medulla, albumin positive staining was localized to blood vessels and small absorption droplets within tubules. (e) In kidneys following 45 minutes of warm arterial ischemia with 1 hour of reperfusion, albumin staining was ubiquitous in the boundary zone, including in the tubular lumen and cytosol of tubular cells.

We first confirmed that 45 minutes of ischemia resulted in venous obstruction of the rat kidney. Venous obstruction was confirmed by a rapid increase in kidney wet weight following reperfusion, and by the extravasation of blood proteins into the kidney parenchyma. Average wet kidney weight per 100 g total body weight increased from 0.32% ± 0.01% in kidneys without ischemia (*n* = 24) to 0.38% ± 0.02% (*n* = 12) and 0.43% ± 0.01% (*n* = 20) at 1 and 3 hours postischemia (*P* < 0.0001), respectively (Figure 1a). This represented an approximately 33% increase in wet weight of the kidney in the first 3 hours of reperfusion. This increase in kidney weight was associated with a marked color change in the cortical parenchyma. As previously reported,^14^ the parenchyma of kidneys recovering from warm ischemia turned blue, consistent with the extravasation of Evans blue from the vascular compartment (Figure 1b and c). In control kidneys, albumin staining in the cortical medullary boundary zone was light and localized to blood vessels and small absorption droplets (Figure 1d). In contrast, in kidneys following 45 minutes of warm arterial ischemia with 1 hour of reperfusion, albumin staining was ubiquitous in the boundary zone, including in the tubular lumen and cytosol of tubular cells (Figure 1e).

**Figure 2:**
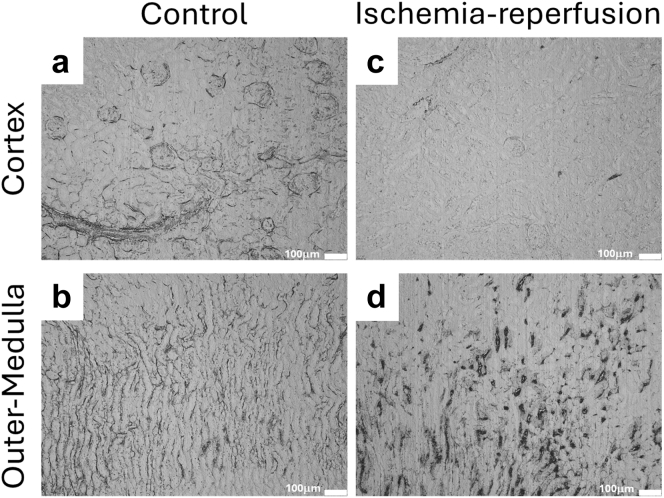
Tracking reperfusion of vessels following reperfusion from renal ischemia using Evans blue. Representative images are shown. In thin sections, extravasated Evans blue–labelled plasma proteins are unseen, whereas vascular staining remains prominent. (a) In control kidneys, the Evans blue stains the glomeruli and peritubular capillaries in the renal cortex. (b) In the outer-medulla of control animals, the vasa recta bundles and peritubular vessels are stained, but congestion is minimal. (c) Following 45 minutes of ischemia and 1 hour of reperfusion, the glomeruli and peritubular capillaries of the cortex remain largely unstained, indicating blood flow was not returned to this region. (d) In the outer medulla of the kidney following 1 hour of reperfusion from warm arterial clamp ischemia, the vasa recta and peritubular capillaries are stained. (d) The stained vasculature is expanded because of the accumulation of red blood cells.

Because these data were consistent with venous blockage and consequent extravasation of large amounts of blood proteins from the congested outer medullary capillaries, we attempted to identify the site of obstruction. To identify the site of obstruction of blood, we tracked the accumulation of RBCs that had entered the kidney after removal of the renal arterial clamp. Following 1 hour of reperfusion of the control kidney, Evans blue–stained RBCs could be observed in the cortical vasculature, glomerular capillaries, outer medullary capillary plexus, vasa recta bundles, and wavy vasa recta (Figure 2a and b). In contrast, and consistent with prolonged cortical vasoconstriction postischemia,^28^ the cortical glomeruli and vasculature of the ischemic kidney remained largely unstained (Figure 2c). In these kidneys, Evans blue–stained RBCs had begun to accumulate in the outer medullary capillary plexus and wavy vasa recta (Figure 2d).

**Figure 3:**
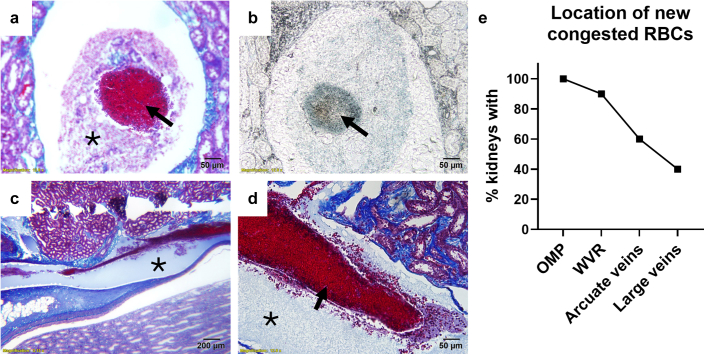
Evidence of vascular obstruction in the large renal veins. (a–e) In some kidneys following 45 minutes of arterial ischemia and 1 hour of reperfusion, tightly packed, congested RBCs could be observed in arcuate and even larger renal venous vessels. (a) Often, this blood was surrounded by an unknown material (∗) containing blood cells. (b) The congested blood, marked by the arrow, was confirmed to be new blood that had entered the kidney during reperfusion because it stained positive for Evans blue. (c and d) Sometimes, the congested blood (arrow) appeared to be obstructed by an amorphous substance (∗), largely devoid of cells, that stained blue with trichrome staining Trichrome-stained images and are shown in (a), (c), and (d). (b) is unstained, with the blue coloring coming from Evans blue. For (a), (b,) and (d), original magnification was 20×. (c) is a low power image (5× original magnification) showing the apparent obstruction of blood in a large segmental vein of the kidney by a blue amorphous substance. (e) is a summary of the % of kidneys (*n* = 10 total, 6 females and 4 males), which had evidence of new, Evans blue–stained, congested RBCs in each vessel type after 45 minutes of ischemia and 1 hour of reperfusion. OMP, outer medullary plexus; RBC, red blood cell; WVR, wavy vasa recta.

Remarkably, in many ischemic kidneys, following 1 hour of reperfusion, Evans blue–stained, congested RBCs, were found in the arcuate and even larger renal veins, suggesting that the obstruction was downstream of these large vessels (Figure 3). We observed that congested RBCs in the large veins of the kidney were surrounded by a homogenous material that stained blue in trichrome sections (Figure 3a–d). Sometimes, this material was largely devoid of cells (Figure 3c and d); however, other times it contained blood cells that did not stain positive for Evans blue, indicating that they were present before the initiation of ischemia (Figure 3a and b). Evans blue–stained congested RBC surrounded by this blue material, were present in the large veins of approximately 40% of kidneys following ischemia with 1 hour of reperfusion (Figure 3e).

**Figure 4:**
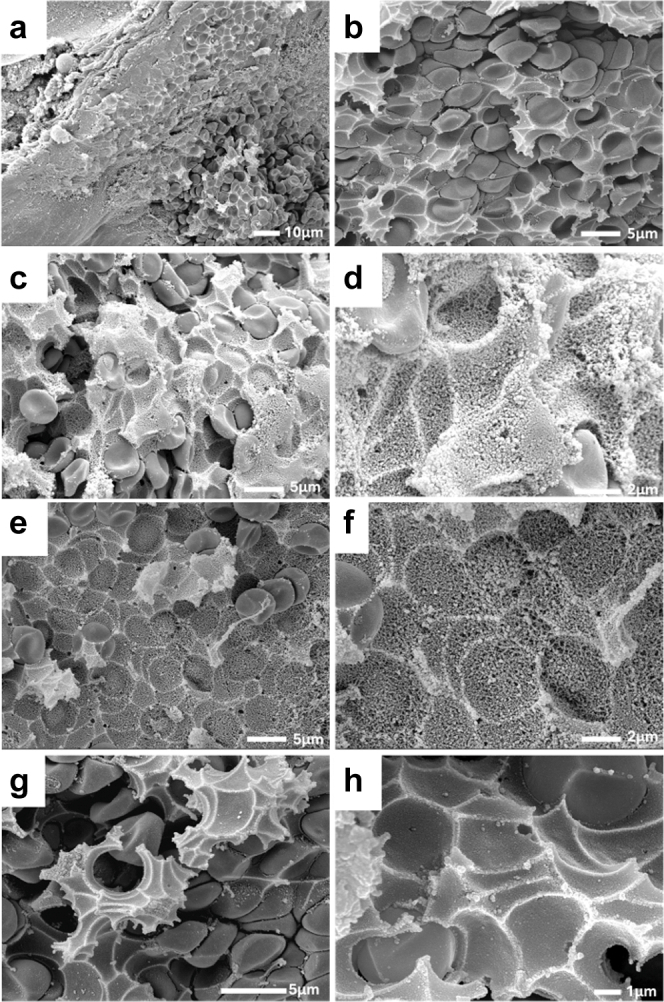
Scanning electron microscopy of congested blood in large renal veins following ischemia. (a–h) Representative scanning electron microscopy images of the large intrarenal veins of the rat kidney after 45 minutes of warm arterial clamp ischemia and 1 hour of reperfusion are shown. Red blood cells are encased by a solid material that appears to hold the shape of blood cells that have been dislodged during processing. (d–f) Sometimes this material appears as a dense network, whereas (g–h) other times it appeared almost completely solid.

Scanning electron microscopy images of the renal veins of ischemic kidneys with 1 hour of reperfusion identified this material as a solid that conformed to and held the shape of entrapped RBCs (Figure 4a–h). Sometimes this material appeared as a matrix of fibers (Figure 4d–f), whereas other times it appeared almost completely solid (Figure 4g and h).

In trichrome-stained sections, similar blue staining “cast” material was commonly found in the renal medulla in predominantly thin limb tubules and vasa recta of postischemic rat kidney sections (Supplementary Figure S2A and B). Blue medullary casts preceded the development of RBC trapping and the formation of red distal tubular casts (Supplementary Figure S2C–G).

**Figure 5:**
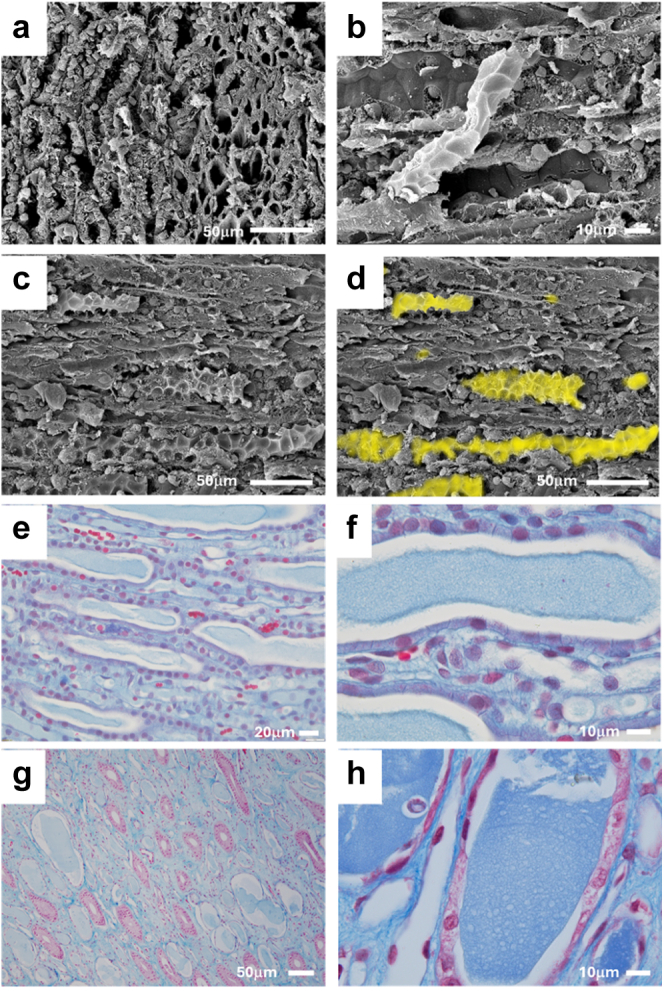
Tubular casts in the papilla of rat and human kidneys. (a) Representative images are shown. Scanning electron micrograph of rat medulla from sham control rat. (a) The tubular and vascular lumens are empty and no casts are observed. (b–d) Scanning electron micrograph of rats after 45 minutes of ischemia and 1 hour of reperfusion. (b) A solid tubular cast appearing to fall out of the lumen on a tubule. (b) The solid casts hold the shape of the collapsed tubular lumen. (c and d) Low power scanning electron micrographs demonstrating prevalence of casts in rat papilla after 1 hour of reperfusion from 45 minutes of warm arterial clamping. (d) Vascular casts and tubular casts from (c) are highlighted in yellow. Images of the rat papilla from trichrome-stained sections of a rat kidney following 3 hours of reperfusion. (e and f) Multiple blue casts are present in papilla. (c–f) Similar to those observed in rats, large, blue staining casts were observed the renal papillary loops of 2 human kidneys. One kidney is shown in (g), the other is shown in (h).

To determine if this blue material may be solid, we performed scanning electron microscopy on the renal papilla of rats following 1 hour of reperfusion from 45 minutes of warm arterial clamp ischemia. Scanning electron microscopy confirmed the presence of solid casts in the lumens of the papillary tubules and capillaries (Figure 5a–d). Similar blue material to that found in the rat papilla postischemia (Figure 5e and f) was prominent in the papilla of the 2 human kidneys (Figure 5g and h).

In many trichrome sections from rats with reperfusion times of 3 hours, blue material was prominent in the outer medullary vascular bundles, juxtamedullary capillary network, the lumens of many proximal tubules, and the Bowman’s space of many juxtamedullary glomeruli (Supplementary Figure S3A–D). When imaged using scanning electron microscopy, this material was found to be solid and resemble that found in the large renal veins (Supplementary Figure S3E–H).

Given that albumin is the most abundant blood protein, we performed immunostaining for albumin (Supplementary Figure S4A and B). The large venous casts stained positive for albumin, however, albumin staining appeared weaker than in many tubular casts (Supplementary Figure S4 Barrow), suggesting that the large venous casts may not consist entirely of concentrated albumin (Supplementary Figure S4A and B).

Mass spectroscopy analysis of laser-dissected material identified albumin and keratins as the major proteins present in rat venous casts, along with some other large blood proteins (Table 1, Supplementary Data File S1). In total, keratins accounted for approximately 56% of total cast proteins identified in rat venous casts. The most common keratin isoforms observed in rat renal venous casts were keratins 10, 6A, 5, and 1 (Table 1). In a control sample made up of kidney tissue dissected outside of the cast area, keratin proteins made up only approximately 3% of the sample. As expected, most proteins identified in the control sample were common cellular proteins (Supplementary Data File S1). Keratins also made up approximately 67% of all proteins found in an outer medullary vascular bundle cast sample (Supplementary Data File S1). Other common proteins found in this sample included albumin and hemoglobin.

**Table 1:** Mass spectrometry

Rat venous cast proteins	Percentage (Based on average PSM)	Also identified in human tubular casts (> 1% psm)
Albumin	15.7	Yes
Keratin, type I cytoskeletal 10^a^	8.81	Yes
Keratin, type II cytoskeletal 6A^a^	8.71	Yes
Keratin, type II cytoskeletal 5^a^	7.69	Yes
Keratin, type II cytoskeletal 1^a^	7.12	Yes
Serotransferrin	3.28	No
Keratin, type II cytoskeletal 7^a^	2.62	Yes
Serine protease 1	2.50	No
Keratin, type II cytoskeletal 2 epidermal^a^	2.2	Yes
Keratin, type I cytoskeletal 14^a^	2.2	Yes
Keratin, type II cytoskeletal 1b^a^	2.10	No
Alpha-1-antiproteinase	1.72	No
Total keratins	64.1	

Table 1 summarizes the mass spectrometry results. Proteins found in rat venous casts are shown in the left column. For rat venous casts, samples were pooled from 2 rats and 3 replicates run using mass spectrometry. Proteins that made up < 1% of the total protein material, or that were not present in all 3 replicates were excluded. This left 12 proteins, 8 of which were keratins (with superscript ^a^). The remaining 4 proteins were common constituents of blood plasma. The % that each protein contributed to the total amount of cast protein based on peptide spectrum match (PSM, including excluded proteins) is shown in the middle column “Percentage.” The right column “Also identified in human tubular cast,” reports whether these proteins were also found to represent > 1% of total tubular cast proteins identified in either of the human kidney samples investigated.

Immunohistochemical staining of postischemic rat kidney sections confirmed the presence of keratins in both the large venous casts and vascular bundles. In rat tissue, vascular staining was light; however, it localized to the anatomical structures in which solid casts had been confirmed by scanning electron microscopy and was absent in samples missing the primary antibody (Supplementary Figure S5A–D).

The large venous casts and vasa recta bundles were positive for keratins 1, 6A, and 10 (Supplementary Figure S6A, B, E–H). Large venous casts were negative for keratin-5 (Supplementary Figure S6C). Other structures to stain positive for keratins 1, 5, 6A, and 10 included the papillary collecting ducts (with staining tending to be strongest toward the papillary tip) and the uroepithelium (Supplementary Figure S7).

Despite anticoagulation with high doses of heparin before arterial clamping, the large venous vessels still contained the material observed in vehicle-treated rats following 45 minutes of kidney ischemia with 1 hour of reperfusion (Supplementary Figure S8A and B).

Next, we examined the localization of keratins 1, 5, 6A, and 10 in sections from human kidneys. In total, tissue from 12 human kidneys was examined. 6 of these had confirmed AKI at the time of kidney harvest. Details for human kidneys are shown in Table 2. Staining of tubular casts was absent in the absence of primary antibody (Supplementary Figure S9A) but was often strong when primary antibody was present (Supplementary Figure 9B). Although some nonspecific staining was identified, primarily around small vascular structures (Supplementary Figure S9C), positive staining in these vessels was often prominent in congested vessels (Supplementary Figure S9D).

**Table 2:** Kidney donor information

Sample	Number	Age, yr	Cr	BUN	Cortical keratin casts	Number of kidneys	Medullary keratin casts	Number of kidneys	ATI	Number of kidneys
No AKI										
Male	4	47 ± 3	0.72 ± 0.13	21 ± 6	Minimal	3	Minimal	2	Minimal	3
Female	2				Some	3	Some	2	Moderate	2
					Common	0	Common	2	Severe	1
With AKI										
Male	3	47 ± 3	4.54 ± 1.06	57 ± 13	Minimal	0	Minimal	3	Minimal	1
Female	3				Some	5	Some	2	Moderate	1
					Common	1	Common	1	Severe	4

AKI, acute kidney injury; ATI, acute tubular injury score; BUN, blood urea nitrogen (mg/dl); Cr, plasma creatinine (mg/dl)

Both BUN and Cr at last measurement before tissue collection.

Data are mean ± SE.

**Figure 6:**
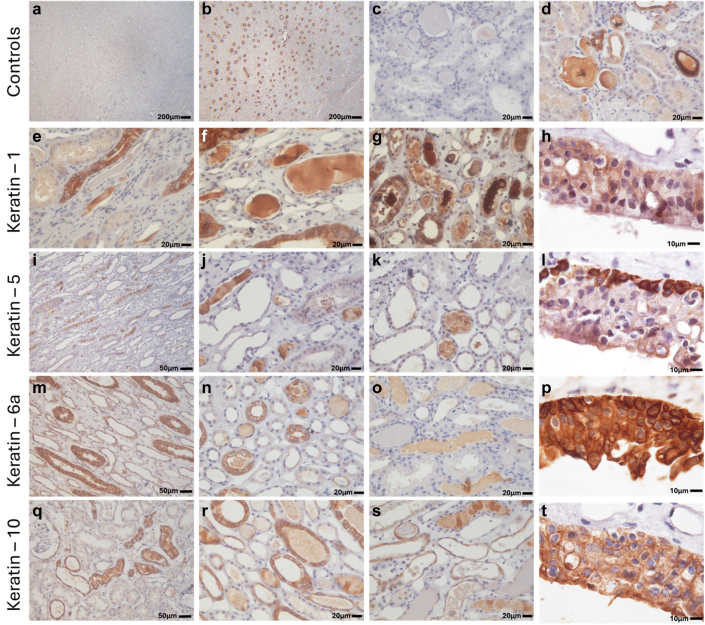
Keratin isoform expression in human donor kidneys. Representative immunohistochemistry images of keratin isoform–specific staining in human donor kidneys. Controls show low power (5× original magnification) images of the renal papilla (a) without and (b) with keratin-1 primary antibody. (b) Collecting ducts stain positive only when primary antibody is present. High power (40× original magnification) images of the renal cortex (c) without and (d) with keratin-1 primary antibody are also shown. (b) Tubular casts stain positive only when primary antibody is present. Keratin-1 staining is shown in (e–h). (e–g) Collecting ducts and many tubular casts, stain strongly positive for keratin-1. (h) The uroepithelium stains strongly positive with diffuse keratin-1 staining across all layers of the epithelium. Keratin-5 staining is shown in (i–l). (I–k) Tubular casts and the uroepithelium stain strongly positive for keratin-5. (l) Keratin-5 staining in the uroepithelium is mostly localized to scattered cells in the basal layer. Keratin-6A staining is shown in (m–p). Collecting ducts as well as other tubular structures and many tubular casts, stain positive for keratin 6A (m–o). (p) The uroepithelium stains strongly positive, with diffuse keratin-6A staining across all layers of the epithelium but most prominent in cells of the basal layer. Keratin-10 staining is shown in (q–t). (q–s) Collecting ducts are strongly positive, but positive staining is also found in other segments of the tubular epithelium. (q–s) Several tubular casts stain positive for keratin-10, whereas others are negative. (t) The uroepithelium stains diffusely positive keratin-10 staining across all layers of the epithelium. In total 12 kidneys were examined.

In human kidneys, keratin staining was prominent in the uroepithelium and collecting ducts (Figure 6). Other segments of the nephron, including proximal tubules and thin limbs stained lightly positive for keratin (Figure 6). Keratin positive tubular casts were present in 10 of 12 human kidney sections examined (Figure 6). Tubular casts in human kidney sections were positive for all of keratin-1 (Figure 6 e–g), keratin-5 (Figure 6i–k), keratin-6A (Figure 6n and o), and keratin-10 (Figure 6q–s). Not all tubular casts, however, stained positive for keratins (Figure 6o and s). Keratin casts were identified in both cortical and medullary tubular segments. In some kidneys, keratin positive casts were identified in the lumens of injured tubules that had detached from the basement membrane. Large keratin casts were also found in the lumens of many dilated dedifferentiated tubules. In 2 kidneys, keratin positive casts were ubiquitous in the papilla, being found in the lumens of thin limbs of the Loop of Henle, collecting ducts, and small vessels (Figure 5g and h).

Consistent with the blue staining cast material in trichrome sections containing keratins, in some human kidneys in which mixed red and blue casts were found in the papilla, only the blue material stained positive for keratins (Supplementary Figure S10A–D). Blue cast material was strongly positive for albumin (Supplementary Figure S10E and F). In contrast, and consistent with degraded hemoglobin as the source of the material found, red cast material was often positive for endogenous peroxidases (Supplementary Figure S10G and H).

Proteomic analysis of tubular cast material isolated from human kidneys identified keratins as a major constituent of the casts. In human kidney tubular casts, keratins and albumin made up 15 of the top 20 most abundant proteins identified in the first cortico-medullary sample, 6 of the top 20 in the second cortico-medullary sample examined, 7 of the top 20 in the first papillary sample investigated, and 1 of the top 20 in the second papillary sample investigated (Supplementary File S1). Keratins and albumin were the most common proteins found in both cortico-medullary human cast samples. In addition to keratin-1, proteins found in both papillary cast samples included albumin, lipocalin-1, lysozyme C, and proline-rich protein-4 (Supplementary File 1).

Three of 12 kidneys from humans were identified to have gross RBC trapping, whereas only 2 of these had AKI at the time of tissue harvest. Tubular casts from these kidneys were confirmed to be positive for keratin-1 (Supplementary Figure S11A) and resembled those found in rats when imaged using electron microscopy (Supplementary Figure S11B and C). Keratin-1 positive vascular casts were identified in these sections (Supplementary Figure S11D–J). RBCs could often be observed surrounded by keratin-1 positive material (Supplementary Figure S11D and H). This material resembled that found in rat veins when imaged using electron microscopy (Supplementary Figure S11E, F, I, and J). Often congested tightly packed RBC appeared to be surrounded by electron dense material (Supplementary Figure S11J).

A large venous cast was observed in only 1 of the 12 human kidneys examined. Similar to those found in rats following ischemia, this large venous cast stained blue with trichrome staining and was positive for both albumin and keratin-1 (Supplementary Figure 12). Because of a lack of sections containing this large venous cast, other keratin isoforms could not be examined.

## Discussion

This study has 2 major findings. First, we find that, in the post-ischemic rat kidney, a solid material obstructs both vascular and tubular lumens. This is an important finding because this material appears to be responsible for the obstruction of venous drainage of the medulla, which results in the development of RBC trapping and subsequent tubular injury.^14^ Second, our data indicates that this material consists largely of albumin and insoluble keratins, including keratin isoforms 1, 6A, and 10. Importantly, we demonstrated that tubular and vascular casts containing the same keratin isoforms are prominent in human donor kidneys.

Vascular obstruction by keratin casts could explain the accumulation of RBCs in the outer medullary circulation of the post-ischemic kidney. We have recently demonstrated that RBC trapping promotes toxic injury to tubular cells secondary to the extravasation of plasma proteins that are then taken up by and cause damage to nearby tubular cells.^14^ Although the accumulation of RBCs in the outer medullary circulation is highly suggestive of obstruction of the vessels that drain the outer medulla, the nature of this obstruction has remained unclear.^8^^,^^9^ It has previously been suggested that RBC trapping occurs because of hypoxic swelling of tubular epithelial cells, which then compress the wavy vasa recta that drain the outer medullary capillary plexus.^9^^,^^29^ This hypothesis is supported by evidence that large doses of mannitol given intravascularly, immediately before clamp ischemia in rats, can limit RBC trapping.^29^ Several observations, however, are not consistent with this hypothesis. Given that during clamp ischemia, the entire kidney parenchyma is hypoxic, tubular swelling should cause generalized RBC trapping in all regions of the kidney. In contrast, we often find that RBC trapping is localized to clearly defined segments of the kidney.^8^^,^^15^ Rather than compression of the wavy vasa recta, this is consistent with the obstruction of larger vessels responsible for regional drainage. Second, increased tubular pressure from ureteral obstruction, which should result in capillary compression, does not cause RBC trapping.^29^ Our finding that vascular obstruction is mediated by a solid material that obstructs blood flow through the renal venous vessels following kidney ischemia can explain these inconsistencies.

Keratins are proteins that form intermediate filaments with the cytosol and are highly insoluble.^30^ Although generally localized to epithelial tissues, some keratins, such as keratin-1, may be expressed in nonepithelial tissues such as endothelial cells.^31^ Our data shed light on the differing nature and timing of the appearance of tubular casts in the rat kidney following ischemia. It is well-established that tubular casts containing heme proteins are often found in the distal tubules following ischemic AKI.^32^^,^^33^ Our data suggest that these casts, which stain positive for endogenous peroxidases,^14^ appear red in trichrome-stained sections. These likely represent the extravasation of heme from degraded RBCs in the congested outer medullary capillary network. In addition, our current data may shed light on the origin and nature of blue tubular and vascular casts. In trichrome sections, we find that blue casts appear earlier than red casts, first appearing in the outer medullary vascular bundles and papilla during the period of ischemia. Importantly, our data suggest that these blue tubular and vascular casts may consist largely of albumin and insoluble keratins. Although the source of these keratins remains unclear, the presence of keratin casts in the lumens of injured tubules suggests that keratins may be leaking from damaged or stressed renal epithelial cells and combining with blood proteins to form the casts.

To the best of our knowledge, the expression of keratins 1, 5, 6A, and 10, which were identified in our study, have not been previously investigated in the kidney. Djudjaj *et al.*^34^ have previously reported keratin expression in almost all tubular epithelial cells of the kidney as well as in parietal cells of the Bowman’s capsule; however, the isoforms investigated in their study, including keratins 7, 8, 18, and 19, differ from the ones we found. Although our study was not designed to detect the cellular sources of the keratins we identified, our data indicate that, like other keratin isoforms examined, these isoforms may be most highly expressed in collecting ducts, thin limbs of the Loop of Henle, and the uroepithelium. Though this expression profile could easily explain keratin positive casts in the collecting ducts, the source of keratin casts in other structures, such as the vasculature remains unclear. Further studies will be needed to identify the source of keratins in this cast material.

Except for Keratin-1, which has been identified as an antigen for antiendothelial antibodies in allograph rejection,^35^ none of the keratins we identified have been reported to be involved in tubular injury or AKI. Other keratin isoforms, however, have been identified as potential biomarkers of tubular injury. Keratins K7, K8, K18, and K19 have been reported to be upregulated in injured tubular cells in both humans and rats, including in AKI.^34^ Interestingly, in regard to our own data, Djudjaj *et al.*^34^ found that in kidneys from patients with multiple myeloma, intratubular casts were positive for keratins 8 and 19. Further, recent studies have reported that urinary levels of cytokeratin 20 may be a sensitive indicator of the severity of AKI and transition of AKI to chronic kidney disease cardiopulmonary bypass.^36^^,^^37^ Although previous studies have identified keratins as potential markers of tubular epithelial cell injury, our study is the first to suggest that keratin casts may be important in driving kidney pathology in AKI by obstructing both tubular and vascular structures.

We have reasonable confidence that the solid casts we identified contain keratin proteins. In addition to keratins, which were unexpected, the other main proteins identified by our mass spectrometry data were consistent with the tissue areas examined. Our mass spectrometry data were reproducible with similar keratin isoforms being identified in all cast-containing samples. Although keratins are often a contaminant in mass spectrometry studies, that they were not a major constituent of our control samples suggest that the keratins we identified are unlikely to be present solely because of contamination. We were able to validate the presence of many of the keratins we identified in renal casts with immunohistochemistry. Although immunohistochemical staining of keratin isoforms in casts from rats was light, it was absent in the absence of a primary antibody. Providing us with greater confidence in our conclusions, staining of keratin isoforms in both tubular and vascular casts in sections from human kidneys was strong. Although our data indicate that keratins 1, 6A, and 10 were the primary isoforms present in the casts we examined, we cannot exclude cross-reactivity of the antibodies we used with other keratin isoforms. There were, however, clear differences in the staining profiles of the antibodies we used across the different layers of the uroepithelium, suggesting some degree of isoform specificity. Given that we did not independently validate the specificity of the antibodies used using knock-out animals, we cannot exclude the possibility that the positive staining we identified was nonspecific. Keratin positive casts, however, were identified with each of the 4 independent antibodies we used; other structures that stained positive using these antibodies are known to contain keratins.

We were able to exclude the possibility that the venous obstructions we observed were traditional fibrin clots. Although platelets and fibrin were sometimes observed in the large veins of rats following ischemia, these did not appear to be a major component of the obstruction; pretreatment with high-dose heparin did not prevent cast formation. These data are consistent with previous reports indicating that fibrin clots are not responsible for RBC trapping.^9^^,^^12^^,^^38^^,^^39^

The occurrence of keratin positive casts in human kidneys examined is reported in Table 2. Despite only investigating 12 human kidneys, 6 with AKI at the time of collection and 6 without, we found similar pathology to that which we had observed in rats in many of the human kidneys we investigated. Keratin positive tubular casts were identified all but 2 of the human kidneys we investigated (both without AKI), albeit the location and number of these casts varied. Vascular casts were less common. Because kidneys were placed on perfusion pumps after procurement, it is possible that some vascular casts may have been dislodged or dissolved before the kidneys were examined. We did, however, find evidence of keratin positive vascular casts surrounding trapped RBCs in the vasa recta of kidneys with gross RBC trapping and AKI, and a large keratin-1 positive venous cast in 1 kidney without AKI. When imaged with scanning electron microscopy, these vascular casts looked remarkably similar to those found in rats; and like in rats, conformed to the shape of the encased RBCs. The presence of keratin positive vascular and tubular casts in human kidneys without AKI is perhaps not surprising. Tubular pathology only loosely associates with the degree of renal functional decline in humans.^40^ Further, our data in rats indicate that keratin positive casts form over the ischemic period. Therefore, human kidney donors without AKI likely followed a similar clinical course to those with AKI, and thus, may have been exposed to periods of renal ischemia. In support of this, half of the kidneys we examined in the group without AKI demonstrated some degree of acute tubular injury.

We speculate that the tubular expression or release of keratins may have both beneficial and deleterious effects on the kidney. Although our data suggest that vascular keratin casts may obstruct venous drainage, leading to RBC trapping and subsequent toxic tubular injury, Yin *et al.*^41^ recently reported that knock-out of keratin-20 in proximal tubular cells is detrimental in AKI in mice. In their study, they found that keratin-20 was rapidly upregulated in proximal tubules following ischemia and that this may protect from tubular cell death from ferroptosis by inhibiting exosomal secretion of antioxidant proteins.^41^ Yin *et al.’s*^41^ data is consistent with the concept that tubular keratin production or release may be an early stress response of the kidney to protect from injury. We speculate that the release of keratin proteins into tubular and vascular lumens may have evolved as a protective mechanism. By obstructing vascular and tubular lumens in an area of injury, keratin release may prevent the spread of toxins or bacteria to unaffected areas of the kidney. Such a mechanism would explain the high levels of keratin expression or release in the papillary tip, because this area is the first region exposed to injury from the infiltration of bacteria from the urinary tract. However, when cell stress is widespread, such as during severe kidney ischemia, it is possible that the mass release of keratins may become deleterious.

In summary, our data provide significantly new insight into the pathogenesis of ischemic AKI. The formation of vascular albumin-keratin casts occurs in the kidney medulla and small and large venous vessels during ischemia. Upon reperfusion, these casts appear to be responsible for post-ischemic venous obstruction, leading to RBC trapping in the outer medullary plexus. As blood accumulates in the medullary capillaries, intravascular pressure increases, driving the extravasation of blood proteins and toxic heme from the now degenerating stagnant RBCs. This results in the formation of “red” distal tubular heme casts and toxic injury to the surrounding tubular epithelium (Supplementary Figure S13). The formation of albumin-keratin casts may represent an important factor in the pathophysiology of ischemic acute tubular injury, which has not previously been recognized.

## Disclosure

All the authors declared no competing interests.

## Supplementary Material

Supplementary Fike (PDF and XLSX) **Figure S1.** Collection of cast material from rat and human kidney sections by laser dissection microscopy. **Figure S2.** Quantification of red and blue tubular casts in rat kidneys following a period of warm ischemia. **Figure S3.** Solid casts in other kidney structures in the rat kidney postischemia. **Figure S4.** Rat large venous cast albumin staining. **Figure S5.** Representative keratin-10 staining in rat kidney sections 1-hour post–ischemia-reperfusion. **Figure S6.** Keratin isoform staining of rat vascular casts. **Figure S7.** Other keratin positive structures in the rat kidney. **Figure S8.** Heparin pretreatment does not prevent solid vascular casts from forming in the rat kidney. **Figure S9.** Representative keratin-1 staining in human kidney sections. **Figure S10.** Red and blue casts in human kidneys. **Figure S11.** Vascular and tubular casts in donor kidneys with AKI. **Figure S12.** Albumin-keratin staining of a large vascular cast from a human kidney. **Figure S13.** Summary figure outlining hypothesis. **File S1.** Proteomic analysis (XLSX). ARRIVE Checklist.

## Footnotes

Supplementary Fike (PDF and XLSX)
**Figure S1.** Collection of cast material from rat and human kidney sections by laser dissection microscopy. **Figure S2.** Quantification of red and blue tubular casts in rat kidneys following a period of warm ischemia. **Figure S3.** Solid casts in other kidney structures in the rat kidney postischemia. **Figure S4.** Rat large venous cast albumin staining. **Figure S5.** Representative keratin-10 staining in rat kidney sections 1-hour post–ischemia-reperfusion. **Figure S6.** Keratin isoform staining of rat vascular casts. **Figure S7.** Other keratin positive structures in the rat kidney. **Figure S8.** Heparin pretreatment does not prevent solid vascular casts from forming in the rat kidney. **Figure S9.** Representative keratin-1 staining in human kidney sections. **Figure S10.** Red and blue casts in human kidneys. **Figure S11.** Vascular and tubular casts in donor kidneys with AKI. **Figure S12.** Albumin-keratin staining of a large vascular cast from a human kidney. **Figure S13.** Summary figure outlining hypothesis. **File S1.** Proteomic analysis (XLSX). ARRIVE Checklist.

## References

[1] Hoffman W.S., Marshall D. (1949). Management of lower nephron nephrosis; report of six cases. Arch Intern Med (Chic).

[2] Lucke B. (1946). Lower nephron nephrosis; the renal lesions of the crush syndrome, of burns, transfusions, and other conditions affecting the lower segments of the nephrons. Mil Surg.

[3] Moeckel G.W., Kashgarian M., Racusen L.C., Jennette J.C., Olson J.L., Silva F.G., D’Agati V.D. (2015).

[4] Ray S.C., Mason J., O’Connor P.M. (2019). Ischemic renal injury: can renal anatomy and associated vascular congestion explain why the medulla and not the cortex is where the trouble starts?. Semin Nephrol.

[5] Bywaters E.G., Dible J.H. (1942). The renal lesion in traumatic anuria. J Pathol Bacteriol.

[6] Reinhard B. (1850). Zur Kenntnis der Brightschen Krankheit. Charité Ann.

[7] Kessel I., Pepler W.J. (1955). Lower nephron nephrosis in the newborn. J Obstet Gynaecol Br Emp.

[8] McLarnon S.C., Johnson C., Giddens P., O’Connor P.M. (2022). Hidden in plain sight: does medullary red blood cell congestion provide the explanation for ischemic acute kidney injury?. Semin Nephrol.

[9] McLarnon S.R. (2023). Pathophysiology of red blood cell trapping in ischemic acute kidney injury. Compr Physiol.

[10] Mason J., Torhorst J., Welsch J. (1984). Role of the medullary perfusion defect in the pathogenesis of ischemic renal failure. Kidney Int.

[11] Lerolle N., Nochy D., Guerot E. (2010). Histopathology of septic shock induced acute kidney injury: apoptosis and leukocytic infiltration. Intensive Care Med.

[12] Mason J., Welsch J., Torhorst J. (1987). The contribution of vascular obstruction to the functional defect that follows renal ischemia. Kidney Int.

[13] Hellberg P.O., Bayati A., Kallskog O., Wolgast M. (1990). Red cell trapping after ischemia and long-term kidney damage. Influence of hematocrit. Kidney Int.

[14] McLarnon S.R., Johnson C., Sun J. (2023). Extravasation of blood and blood toxicity drives tubular injury from RBC trapping in ischemic AKI. Function (Oxf).

[15] McLarnon S.R., Wilson K., Patel B. (2022). Lipopolysaccharide Pretreatment Prevents Medullary Vascular Congestion following Renal ischemia by Limiting Early reperfusion of the Medullary Circulation. J Am Soc Nephrol.

[16] Yamamoto K., Wilson D.R., Baumal R. (1984). Outer medullary circulatory defect in ischemic acute renal failure. Am J Pathol.

[17] Crislip G.R., Patel B., Mohamed R. (2020). Ultrasound measurement of change in kidney volume is a sensitive indicator of severity of renal parenchymal injury. Am J Physiol Ren Physiol.

[18] Crislip G.R., O’Connor P.M., Wei Q., Sullivan J.C. (2017). Vasa recta pericyte density is negatively associated with vascular congestion in the renal medulla following ischemia reperfusion in rats. Am J Physiol Ren Physiol.

[19] Lortie M., Gauthier B., Plante G.E. (1994). Renal reperfusion injury: sequential changes in function and regional albumin extravasation. Microvasc Res.

[20] Wick M.J., Harral J.W., Loomis Z.L., Dempsey E.C. (2018). An optimized Evans Blue protocol to assess vascular leak in the mouse. J Vis Exp.

[21] Honeycutt S.E., O’Brien L.L. (2021). Injection of Evans Blue dye to fluorescently label and image intact vasculature. BioTechniques.

[22] Coscia F., Doll S., Bech J.M. (2020). A streamlined mass spectrometry-based proteomics workflow for large-scale FFPE tissue analysis. J Pathol.

[23] Kuppuswamy S., Watson N.J., Ledford W.L. (2025). Brain proteome changes after intracerebral hemorrhage in aged male and female mice. Neurobiol Dis.

[24] Zhu W., Smith J.W., Huang C.M. (2010). Mass spectrometry-based label-free quantitative proteomics. J Biomed Biotechnol.

[25] Liem H.H., Cardenas F., Tavassoli M., Poh-Fitzpatrick M.B., Muller-Eberhard U. (1979). Quantitative determination of hemoglobin and cytochemical staining for peroxidase using 3,3',5,5'-tetramethylbenzidine dihydrochloride, a safe substitute for benzidine. Anal Biochem.

[26] Bankhead P., Loughrey M.B., Fernandez J.A. (2017). QuPath: open source software for digital pathology image analysis. Sci Rep.

[27] Yunker L., Harwig M.C., Kriegel A.J. (2025). A novel automated method for comprehensive renal cast quantification from rat kidney sections using QuPath. Am J Physiol Ren Physiol.

[28] Oken D.E. (1984). Hemodynamic basis for human acute renal failure (vasomotor nephropathy). Am J Med.

[29] Mason J., Joeris B., Welsch J., Kriz W. (1989). Vascular congestion in ischemic renal failure: the role of cell swelling. Miner Electrolyte Metab.

[30] Moll R., Franke W.W., Schiller D.L., Geiger B., Krepler R. (1982). The catalog of human cytokeratins: patterns of expression in normal epithelia, tumors and cultured cells. Cell.

[31] Remotti F., Fetsch J.F., Miettinen M. (2001). Keratin 1 expression in endothelia and mesenchymal tumors: an immunohistochemical analysis of normal and neoplastic tissues. Hum Pathol.

[32] Oliver J., Mac D.M., Tracy A. (1951). The pathogenesis of acute renal failure associated with traumatic and toxic injury; renal ischemia, nephrotoxic damage and the ischemic episode. J Clin Invest.

[33] Brun C., Munck O. (1957). Lesions of the kidney in acute renal failure following shock. Lancet.

[34] Djudjaj S., Papasotiriou M., Bulow R.D. (2016). Keratins are novel markers of renal epithelial cell injury. Kidney Int.

[35] Guo X., Hu J., Luo W. (2017). Analysis of sera of recipients with allograft rejection indicates that keratin 1 is the target of anti-endothelial antibodies. J Immunol Res.

[36] Ma R., Ouyang H., Chen C. (2025). Urinary cytokeratin 20 predicts severe acute kidney injury and major adverse kidney events in adults undergoing cardiac surgery. Am J Nephrol.

[37] Ouyang H., Ma R., Yang X. (2025). Urinary cytokeratin 20 as a biomarker for AKI-CKD transition among patients with acute decompensated heart failure and acute kidney injury. J Am Soc Nephrol.

[38] Enestrom S., Druid H., Rammer L. (1988). Fibrin deposition in the kidney in post-ischaemic renal damage. Br J Exp Pathol.

[39] Summers W.K., Jamison R.L. (1971). The no reflow phenomenon in renal ischemia. Lab Investig.

[40] Finckh E.S., Jeremy D., Whyte H.M. (1962). Structural renal damage and its relatin to clinical features in acute oliguric renal failure. Q J Med.

[41] Yin L., Deng Z., Liu J. (2025). KRT20 suppresses exosomal secretion of PRDX2 and ferroptosis in acute kidney injury. J Am Soc Nephrol.

