# Supplementary material for: Albumin-Keratin Casts Obstruct Renal Tubular and Vascular Lumens Following Kidney Ischemia
Source: Kidney Int Rep. 2026 Feb 12;11(4):106356. doi: 10.1016/j.ekir.2026.106356 (PMC12996993; doi:10.1016/j.ekir.2026.106356)
Supplement: Supplementary Fike (PDF and XLSX) — Figure S1. Collection of cast material from rat and human kidney sections by laser dissection microscopy. Figure S2. Quantification of red and blue tubular casts in rat kidneys following a period of warm ischemia. Figure S3. Solid casts in other kidney structures in the rat kidney postischemia. Figure S4. Rat large venous cast albumin staining. Figure S5. Representative keratin-10 staining in rat kidney sections 1-hour post–ischemia-reperfusion. Figure S6. Keratin isoform staining of rat vascular casts. Figure S7. Other keratin positive structures in the rat kidney. Figure S8. Heparin pretreatment does not prevent solid vascular casts from forming in the rat kidney. Figure S9. Representative keratin-1 staining in human kidney sections. Figure S10. Red and blue casts in human kidneys. Figure S11. Vascular and tubular casts in donor kidneys with AKI. Figure S12. Albumin-keratin staining of a large vascular cast from a human kidney. Figure S13. Summary figure outlining hypothesis. File S1. Proteomic analysis (XLSX). ARRIVE Checklist. [file mmc1.pdf]

NOTE: Please save this file locally before filling in the table, DO NOT work on the file within your internet browser as changes will not be saved. Adobe Acrobat Reader (available free here) is recommended for completion.

# The ARRIVE guidelines 2.0: author checklist

## The ARRIVE Essential 10

These items are the basic minimum to include in a manuscript. Without this information, readers and reviewers cannot assess the reliability of the findings.

| Item | | Recommendation | Section/line number, or reason for not reporting |
|---|---|---|---|
| **Study design** | 1 | For each experiment, provide brief details of study design including:<br>a. The groups being compared, including control groups. If no control group has been used, the rationale should be stated.<br>b. The experimental unit (e.g. a single animal, litter, or cage of animals). | a. Methods, b. figure legends |
| **Sample size** | 2 | a. Specify the exact number of experimental units allocated to each group, and the total number in each experiment. Also indicate the total number of animals used.<br>b. Explain how the sample size was decided. Provide details of any *a priori* sample size calculation, if done. | a. All n described in results and figure legends |
| **Inclusion and exclusion criteria** | 3 | a. Describe any criteria used for including and excluding animals (or experimental units) during the experiment, and data points during the analysis. Specify if these criteria were established *a priori*. If no criteria were set, state this explicitly.<br>b. For each experimental group, report any animals, experimental units or data points not included in the analysis and explain why. If there were no exclusions, state so.<br>c. For each analysis, report the exact value of *n* in each experimental group. | a. Statistics<br>b. statistics<br>c. Resutls and figure legends |
| **Randomisation** | 4 | a. State whether randomisation was used to allocate experimental units to control and treatment groups. If done, provide the method used to generate the randomisation sequence.<br>b. Describe the strategy used to minimise potential confounders such as the order of treatments and measurements, or animal/cage location. If confounders were not controlled, state this explicitly. | Statistics<br>Statistics |
| **Blinding** | 5 | Describe who was aware of the group allocation at the different stages of the experiment (during the allocation, the conduct of the experiment, the outcome assessment, and the data analysis). | Statistics |
| **Outcome measures** | 6 | a. Clearly define all outcome measures assessed (e.g. cell death, molecular markers, or behavioural changes).<br>b. For hypothesis-testing studies, specify the primary outcome measure, i.e. the outcome measure that was used to determine the sample size. | Methods<br>Methods |
| **Statistical methods** | 7 | a. Provide details of the statistical methods used for each analysis, including software used.<br>b. Describe any methods used to assess whether the data met the assumptions of the statistical approach, and what was done if the assumptions were not met. | Statistics<br>Statistics |
| **Experimental animals** | 8 | a. Provide species-appropriate details of the animals used, including species, strain and substrain, sex, age or developmental stage, and, if relevant, weight.<br>b. Provide further relevant information on the provenance of animals, health/immune status, genetic modification status, genotype, and any previous procedures. | Methiods/animals Table 2.<br>Methods |
| **Experimental procedures** | 9 | For each experimental group, including controls, describe the procedures in enough detail to allow others to replicate them, including:<br>a. What was done, how it was done and what was used.<br>b. When and how often.<br>c. Where (including detail of any acclimatisation periods).<br>d. Why (provide rationale for procedures). | Methods |
| **Results** | 10 | For each experiment conducted, including independent replications, report:<br>a. Summary/descriptive statistics for each experimental group, with a measure of variability where applicable (e.g. mean and SD, or median and range).<br>b. If applicable, the effect size with a confidence interval. | Results<br>Figure legends |

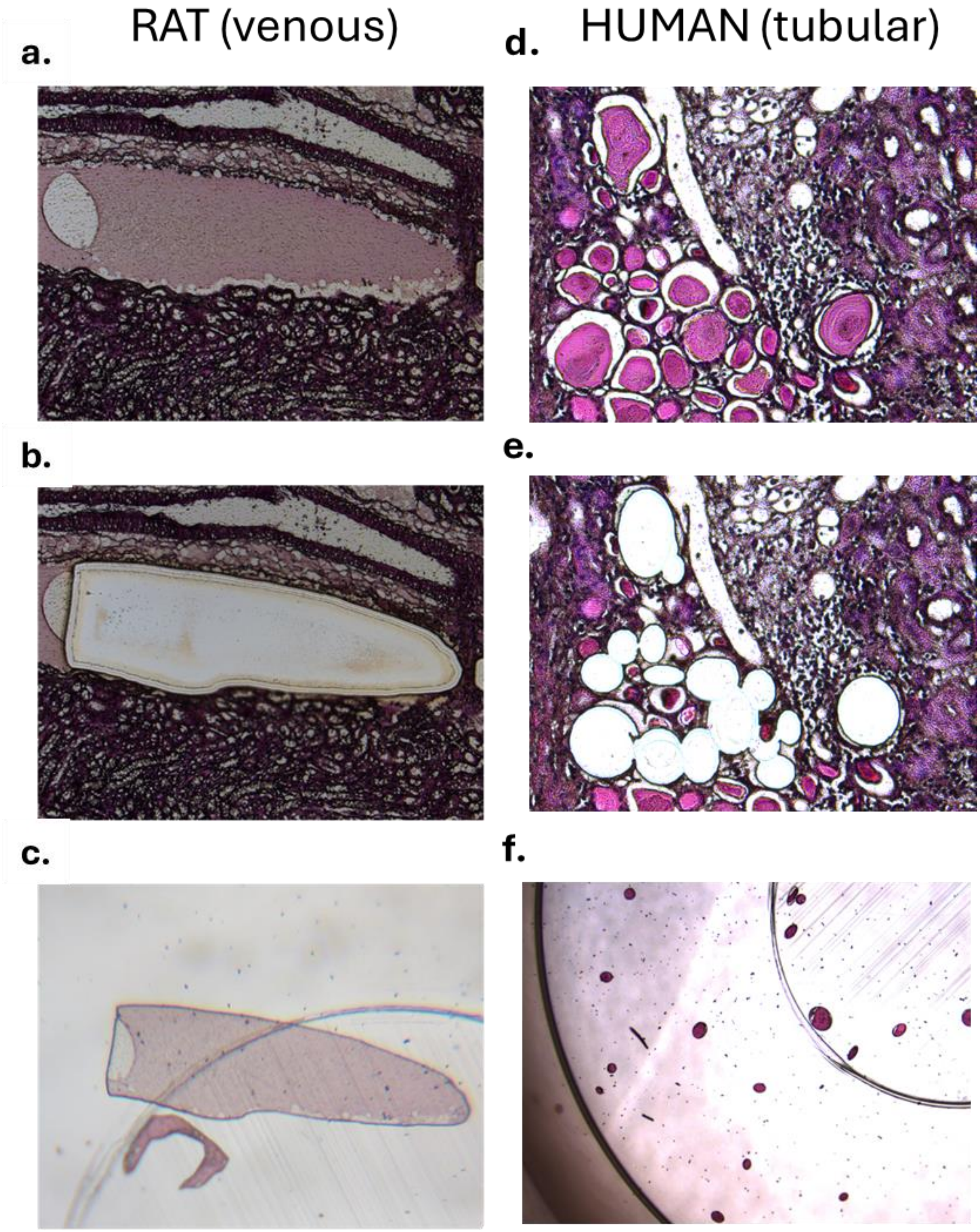


**Supplementary Figure 1**. **Collection of cast material from rat and human kidney sections by Laser dissection microscopy.** Laser dissection microscopy was utilized to collect vascular and tubular cast material from hematoxylin and eosin-stained paraffin sections. For rats, sections of cell free cast material were isolated from large veins (**a-b**), pooled, and collected into protein-compatible

dry collection tube caps (**c**). For human tissue, tubular casts were dissected from individual patient samples (**d-f**)

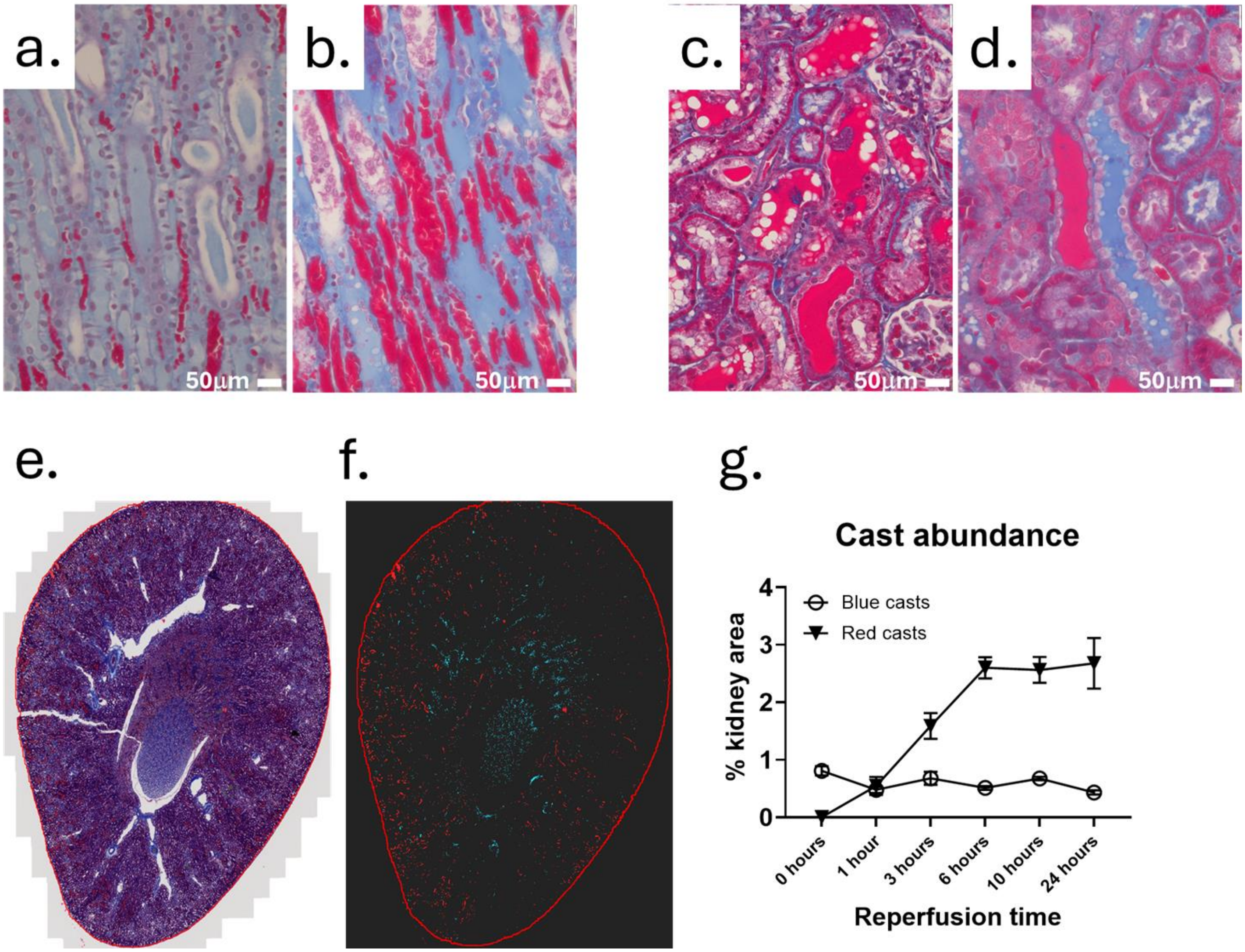


**Supplementary Figure 2. Quantification of red and blue tubular casts in rat kidneys following a period of warm ischemia.** Representative images shown. Trichrome stained image showing blue casts in rat papilla (**a**). Trichrome stained image showing blue casts in rat outer-medullary vascular bundle (**b**). Trichrome stained image showing red casts in rat cortex (**c**). Trichrome stained image showing both red and blue casts in rat cortex (**d**). A digitized trichrome stained section of whole rat kidney (**e**). Automated image analysis of localization of red and blue colored casts in the kidney 1-hour post-reperfusion (**f**). Red casts are developing predominantly in the cortex while blue casts are prominent in the papilla and outer-medulla (**f**). Figure shows results of image quantification at time points from 0 hours (end 45 minutes of ischemia) to 24 hours of reperfusion following 45 minutes of warm arterial clamp ischemia (**g**). Analysis was performed on slides from a previously published time

course study (Mclarnon, JASN 2022). n=4/6 male rats per group. Data are expressed as mean±SE of cast area as a % of total kidney area. Open circles, blue casts. Closed triangles, red casts.

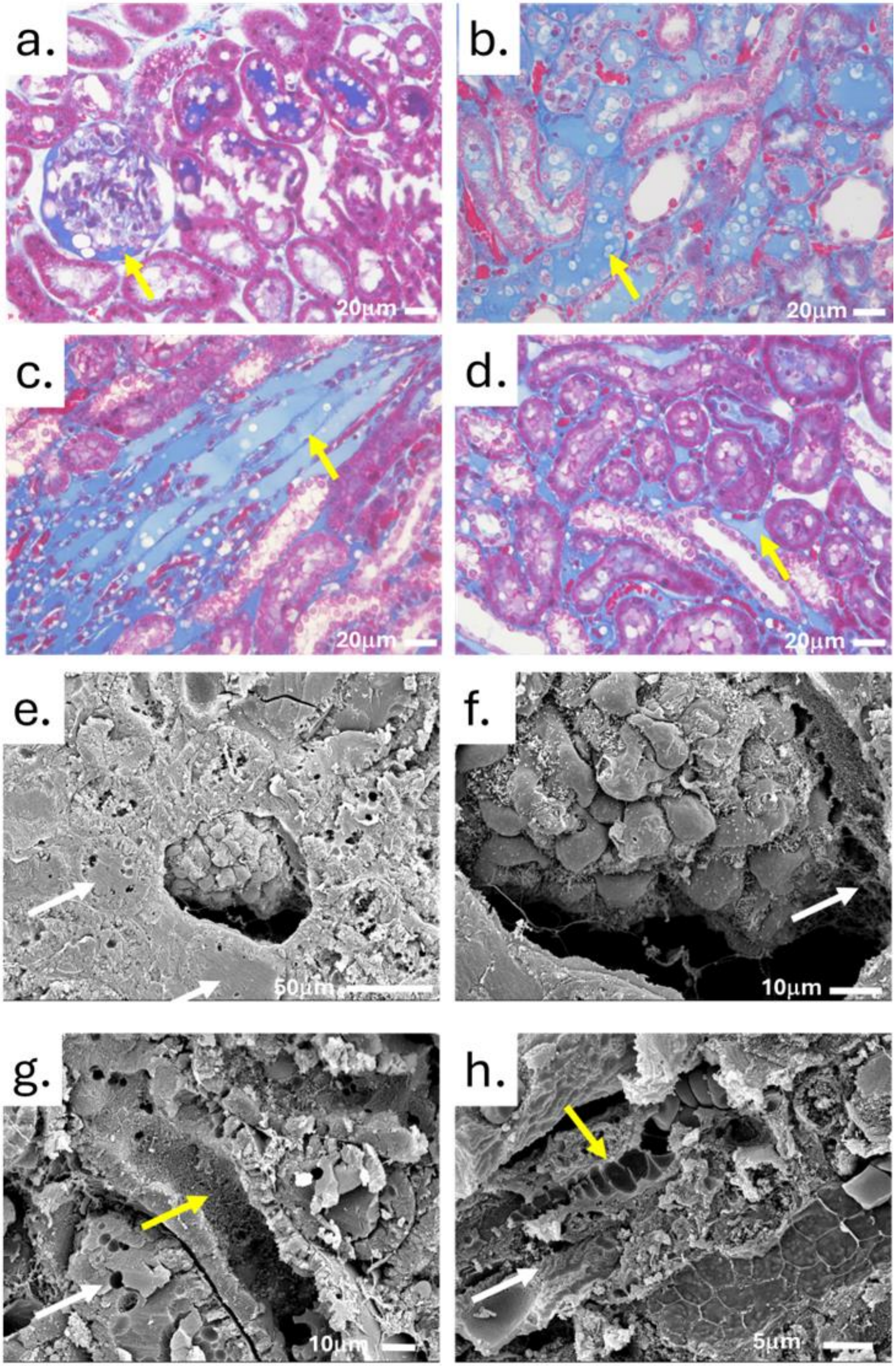
a.
20μm
b.
20μm
c.
20μm
d.
20μm
e.
50μm
f.
10μm
g.
10μm
h.
5μm

**Supplementary Figure 3. Solid casts in other kidney structures in the rat kidney post-ischemia.** Representative images are shown. Panels (**a-d**), are representative trichrome sections from a rat kidney following 3-hours of reperfusion from 45 minutes of warm arterial clamping. Blue 'cast' material is observed in the lumens of proximal tubules and the Bowman's space of a glomeruli (**arrows**) at the cortical-medullary border (**a-b**). Blue material appears to fill the lumens of most tubules at the cortical-medullary boundary zone (**f**). Blue material fills a vascular bundle and surrounding capillary structures (**g**). Red blood cells in the peritubular capillaries can be seen to be surrounded by blue cast material at the cortical-medullary border (**c-d, arrows**). Panels (**e-f**) are scanning electron microscopy images. The lumens of many tubules in the cortical medullary region (white **arrows**) appear to be obstructed by a solid material (**e, g**), A mesh like substance, similar to that observed in the lumens of rat large veins following ischemia, is also present in the Bowman's space of a glomeruli (**f**). Two distinct casts are observed in the lumens of tubules at the cortical-medullary border (**g**). One (**g, white arrow**) appears solid, while the other (**g, yellow arrow**), appears as a mesh of fibers. Two solid casts protrude from the ends of tubular structures (**h, white arrow**). Similar cast material appears have conformed to the shape of RBCs within a capillary vessel (**h, yellow arrow**).

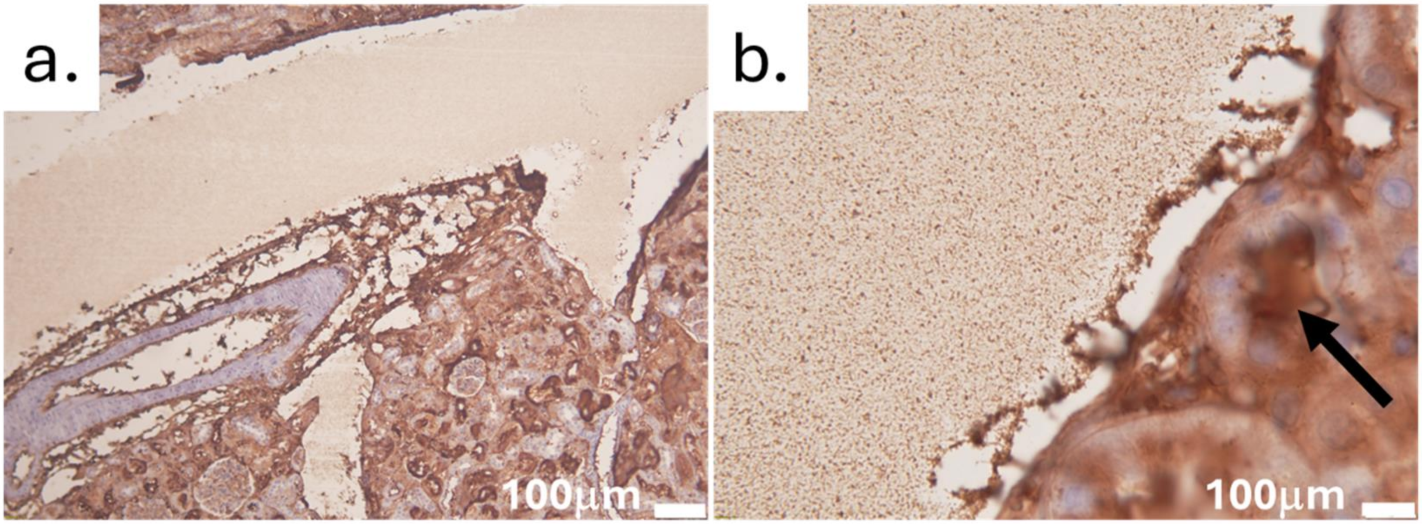


**Supplementary Figure 4. Rat large venous cast albumin staining.** Representative images of large venous casts in rat kidneys following 45 minutes of warm arterial ischemia with 1 hour of reperfusion stained with anti-primary albumin antibody are shown. Venous casts stained mildly positive for albumin (**a-b**). Under high power (100X) magnification, albumin staining was granular in appearance and was less than that observed in many tubular casts (**b, arrow**).

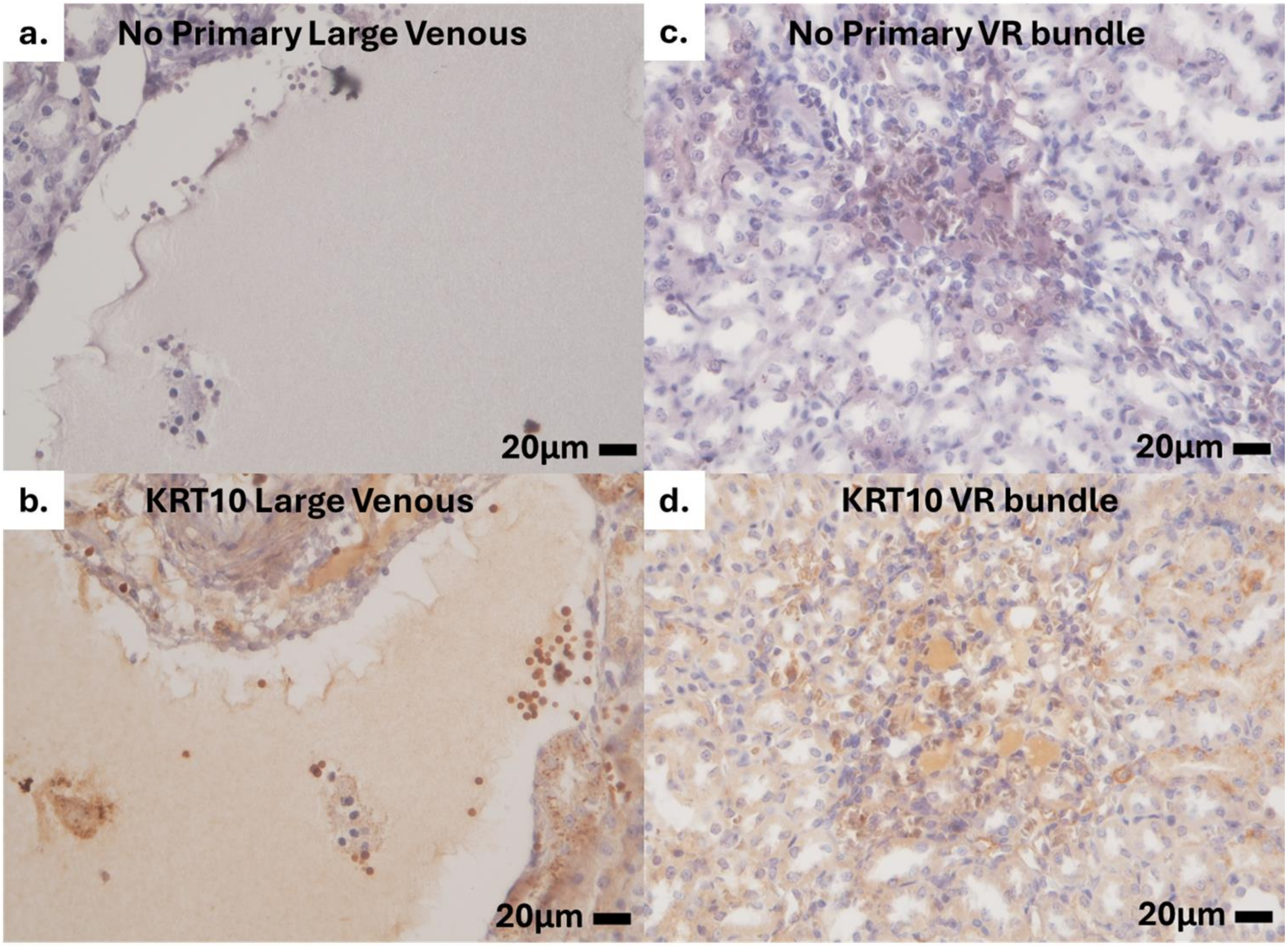


**Supplementary Figure 5. Representative Keratin-10 staining in rat kidney sections 1-hour post-ischemia reperfusion.** Panel **a,** shows a large venous casts absent primary antibody. Positive staining is absent. Panel **b,** shows a large venous casts with primary antibody against Keratin-10. Light positive staining (brown) is present in the cast. Many blood cells in the vein are strongly positive. Panel **c**, shows a vasa recta bundle with 'casts' present absent primary antibody. Positive staining is absent. Panel **d**, shows a vasa recta bundle with 'casts' present with primary antibody against Keratin-10. Casts in vasa recta bundle stain lightly positive for Keratin-10. In total n=10 kidneys were examined.

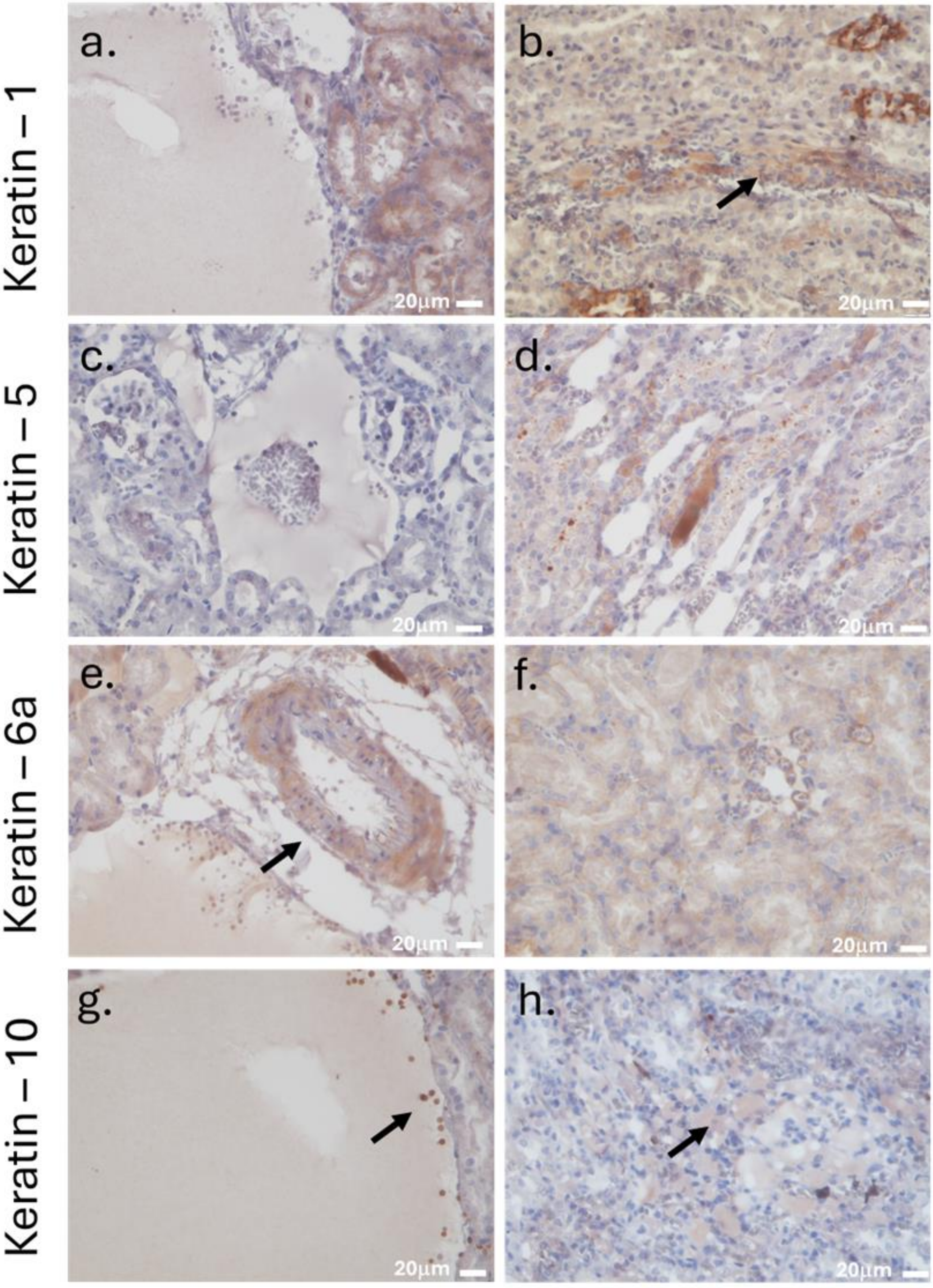
Keratin – 1
Keratin – 5
Keratin – 6a
Keratin – 10
a.
b.
c.
d.
e.
f.
g.
h.
20μm

**Supplementary Figure 6**. **Keratin isoform staining of rat vascular casts.** Representative immunohistochemistry images of keratin isoform specific staining of vascular casts in the rat kidney following 45 minutes of warm arterial clamp ischemia and 1 hour of reperfusion are shown (**a-h**). A large venous cast stains lightly positive for keratin-1 (**a**). Cast material within the vascular bundles of the outer-medulla also stains positive for keratin-1 (**b, arrow**). A venous cast surrounding a core of packed red blood cells is seen. Both stain negative for keratin-5 (**c**). Casts within an outer-medullary vascular bundle stain positive for keratin-5 (**d**). A vascular cast stains positive for keratin-6A (**e**). A nearby arterial wall also stains positive for keratin-6A (**e, arrow**). Proximal tubules as well as parts of the vascular bundle are positive for keratin-6A (**f**). A large venous cast stains positive for keratin-10 (**g**). Blood cells at the edge of the cast also stain positive (**g, arrow**). Vessels within an outer-medullary vascular bundle also stain lightly positive for keratin-10 (**h, arrow**). In total n=10 kidneys were examined.

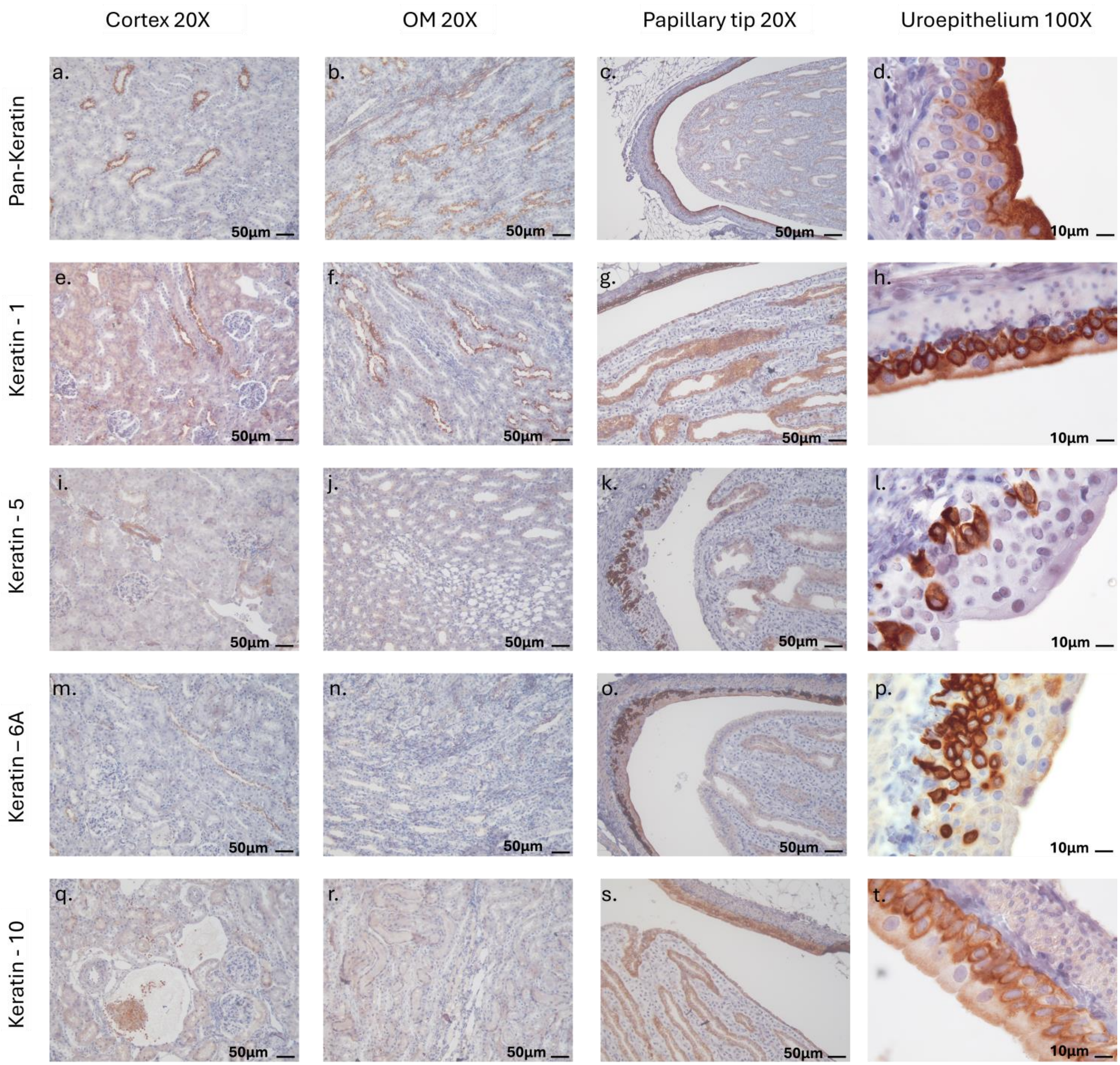


**Supplementary Figure 7**. **Other keratin positive structures in the rat kidney.** Representative immunohistochemistry images of keratin isoform specific staining in the rat kidney are shown. 20X original magnification images of the cortex, outer-medulla (OM) and papilla are shown for each isoform. 100X original magnification images of the uroepithelium are also shown. Pan keratin (**a-d**) stains the collecting ducts, vascular bundles of the OM and uroepithelium. Pan keratin staining of the

uroepithelium is mostly in the superficial layer (**d**). Both cortical and medullary collecting ducts and uroepithelium stain positive for Keratin-1 (**e-g**), with staining in the uroepithelium being most prominent in the basal layer (**h**). Collecting ducts stain lightly positive for Keratin-5 along with arterial walls (**i-k**). Scattered cells in the basal layer of the uroepithelium are positive for keratin-5 (**l**). Cortical collecting ducts and the uroepithelium stain positive for keratin-6A (**m-n**), while there is weak positivity in collecting ducts of the papilla (**o**). Uroepithelial Keratin-6A staining is strongest in the basal layer (**p**). Proximal tubules, some red blood cells, particularly those within venous casts, papillary collecting ducts and the uroepithelium stain positive for Keratin-10 (**q-s**). There is diffuse positive staining across all layers of the uroepithelium with the strongest staining localized to the basal layer (**t**).

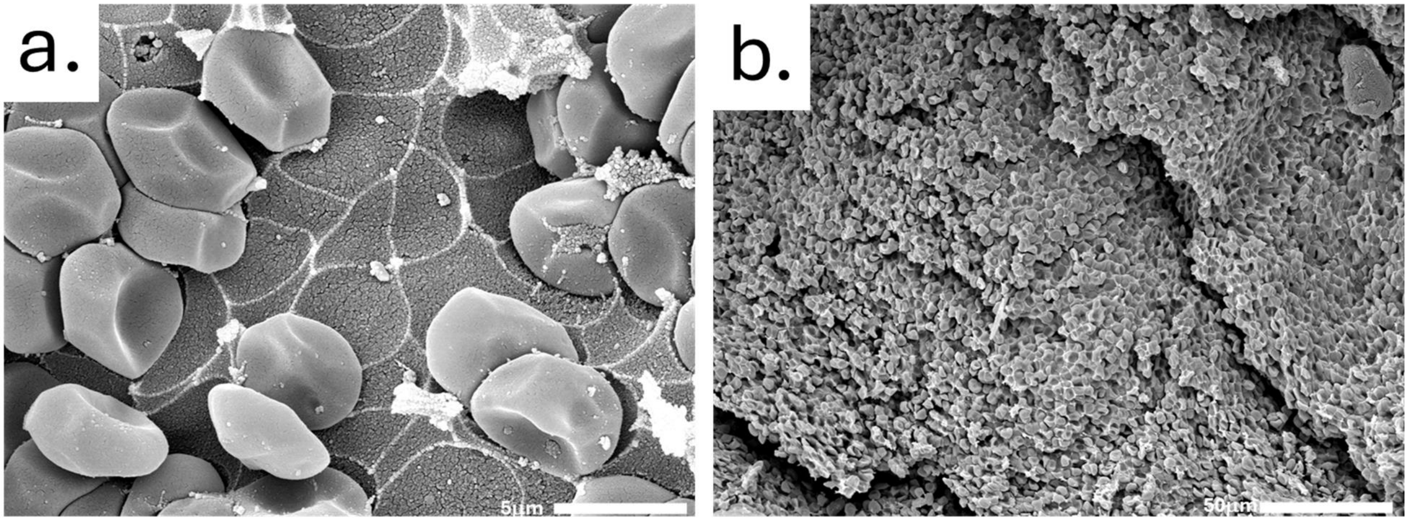


**Supplementary Figure 8. Heparin pre-treatment does not prevent solid vascular casts from forming in the rat kidney.** Representative scanning electron micrographs of large renal vein following 45 minutes of ischemia without reperfusion in the rat kidney are shown. Despite heparin pre-treatment, cast material remains (**a-b**).

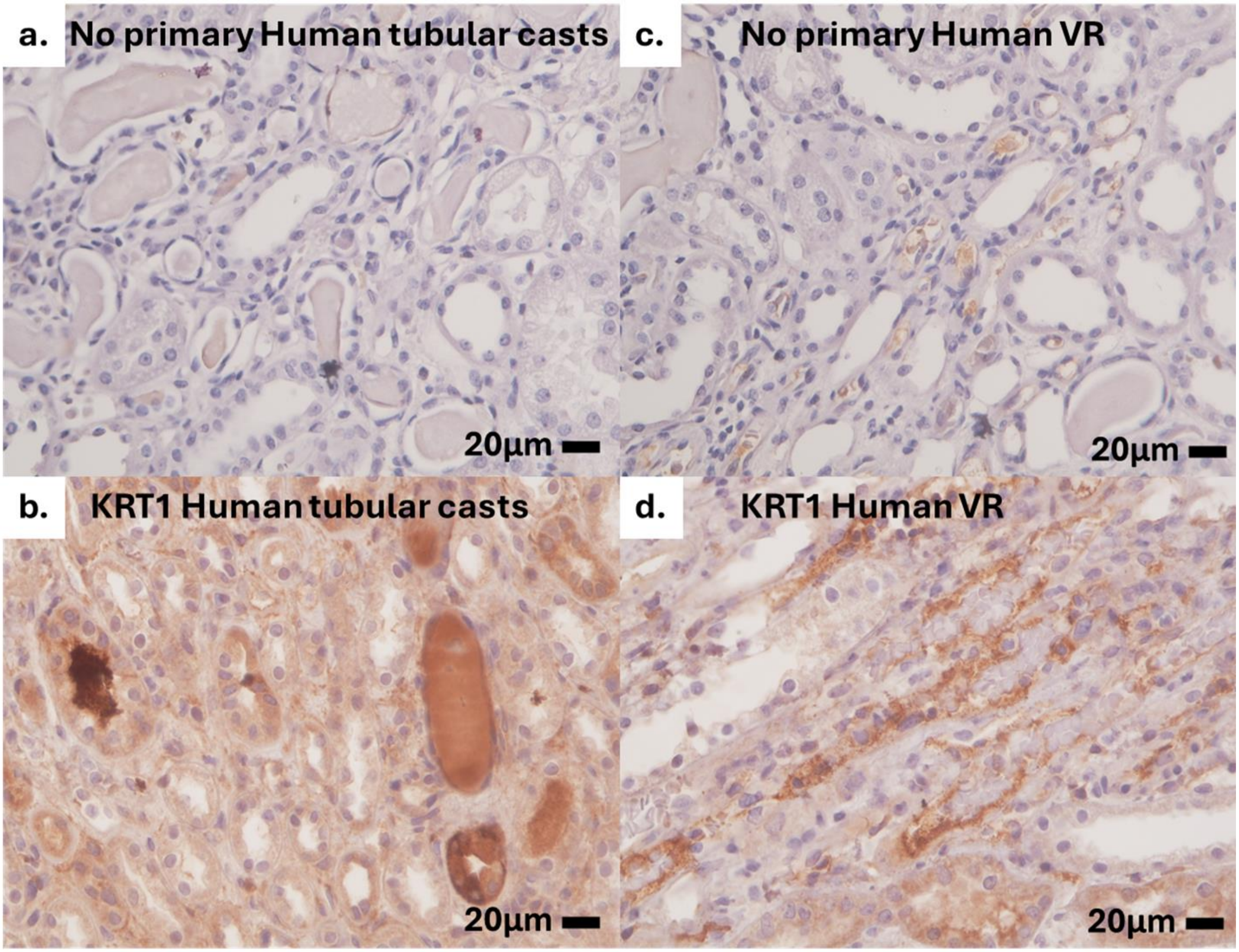


**Supplementary Figure 9. Representative Keratin-1 staining in human kidney sections. Panel a,** shows a section with numerous tubular casts absent primary antibody. Positive staining is absent. **Panel b,** shows a tubular casts with primary antibody against Keratin-1. Many tubular casts are strongly positive for Keratin-1. **Panel c**, shows a vasa recta bundle absent primary antibody. There is some non-specific positive staining in the vessels. **Panel d**, shows a vasa recta bundle from a kidney with AKI and vascular congestion with primary antibody against Keratin-1. Congested red blood cells are surrounded by Keratin-1 positive material. In total n=12 kidneys were examined.

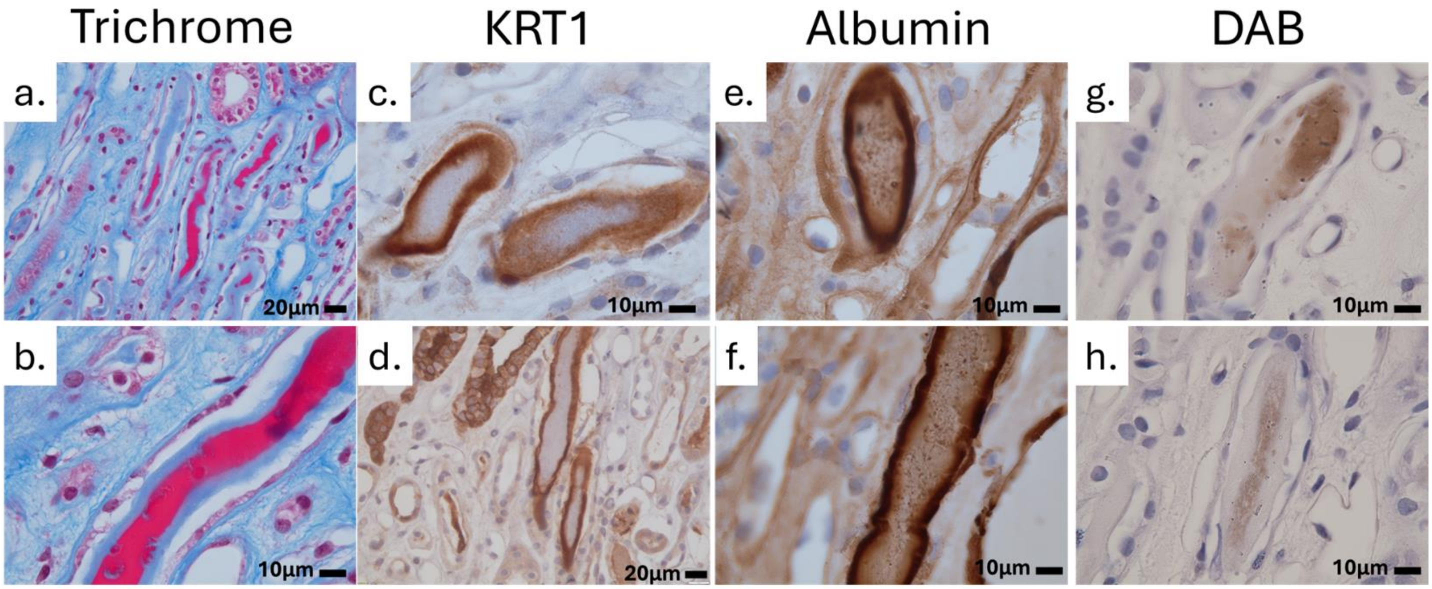


**Supplementary Figure 10. Red and blue casts in human kidneys**. Following trichrome staining, dual colored red and blue casts were found in both rat and human kidneys. Red/blue casts from a single human kidney are shown (**a-h**). With trichrome staining of casts, blue material often surrounds a red core (**a-b**). Only the blue material in the casts stained positive for Keratin-1 (KRT1; **c-d**). Both regions of the cast stain positive for albumin, however, albumin staining is greatest in the outer 'blue' cast material (**e-f**). Often the 'red' core of the casts would stain positive for endogenous peroxidase activity using 3,3'-Diaminobenzidine (DAB), consistent with the presence of hemoglobin (**g-h**).

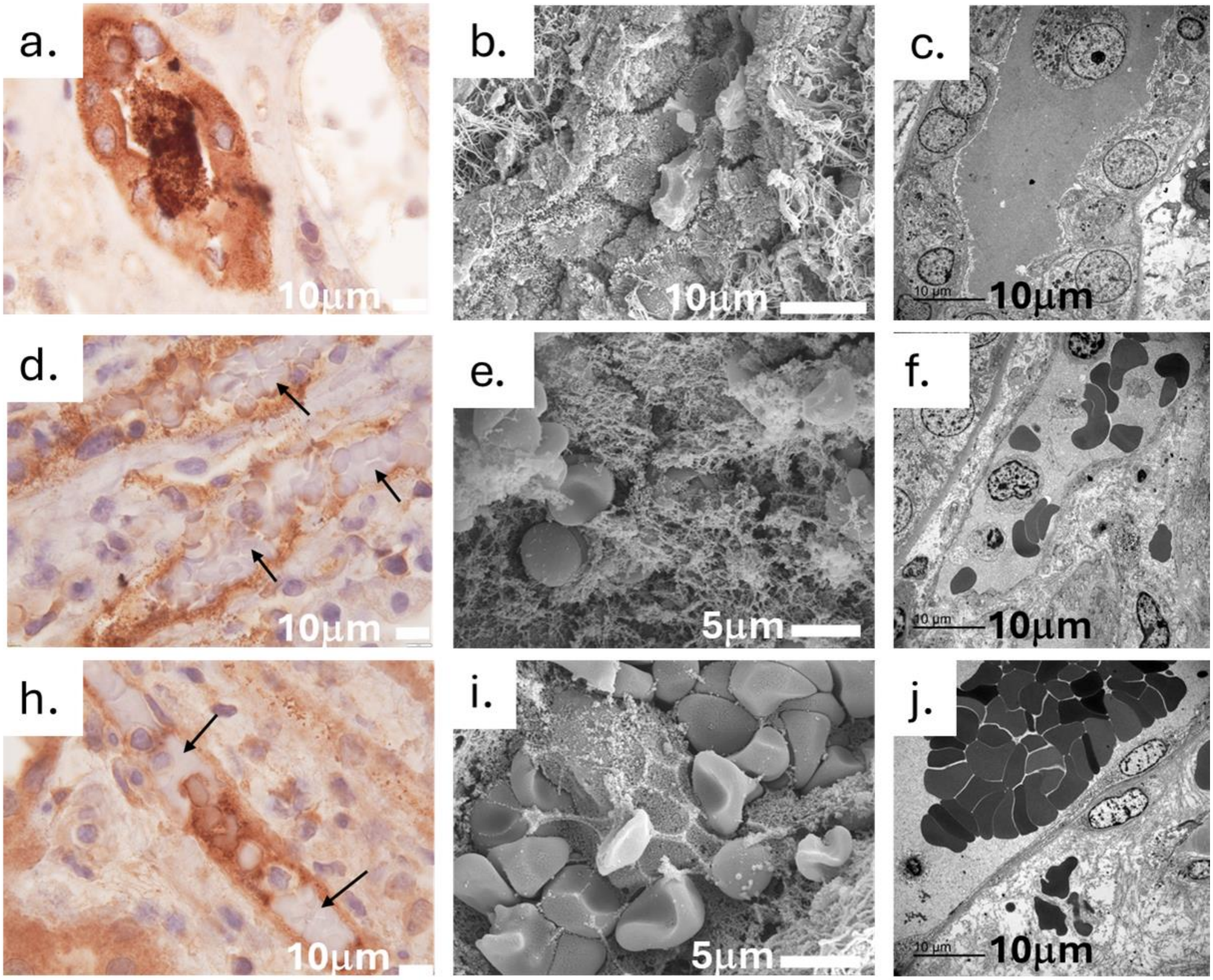


**Supplementary Figure 11. Vascular and tubular casts in donor kidneys with AKI.** Representative images of tubular and vascular casts in human donor kidneys with AKI and gross RBC trapping are shown. A Keratin-1 positive tubular cast (**a**). Scanning electron microscopy images indicate that tubular casts are solid and resemble those observed in rats (**b**). When viewed with transmission electron microscopy, tubular casts appear as dense material in a tubular lumen (**c**). Material surrounding packed red blood cells in congested vasa recta vessels in the outer-medulla also stain positive for Keratin-1 (**d, arrows).** Using scanning electron microscopy, RBC's in an outer-medullary vessel appear to be surrounded by a lose matrix of material (**e**). When imaged using transmission electron microscopy,

RBC's in an outer-medullary blood vessel can be observed to be surrounded by cast material (**f**). High power (100x original magnification) image of a single capillary in which congested RBCs can be seen on either side of Keratin-1 positive material in the vascular lumen (**h**). Scanning electron microscopy image showing tightly packed, congested, red blood cells in an outer-medullary. Material in the vessels looks similar to that found in rats and appears to conform to the shape of the surrounding red blood cells (**i**). Transmission electron microscopy identifies a tightly packed core of red blood cells in an outer-medullary vessel that surrounded by cast material (**j**). In total n=6 kidneys from patients with AKI were examined.

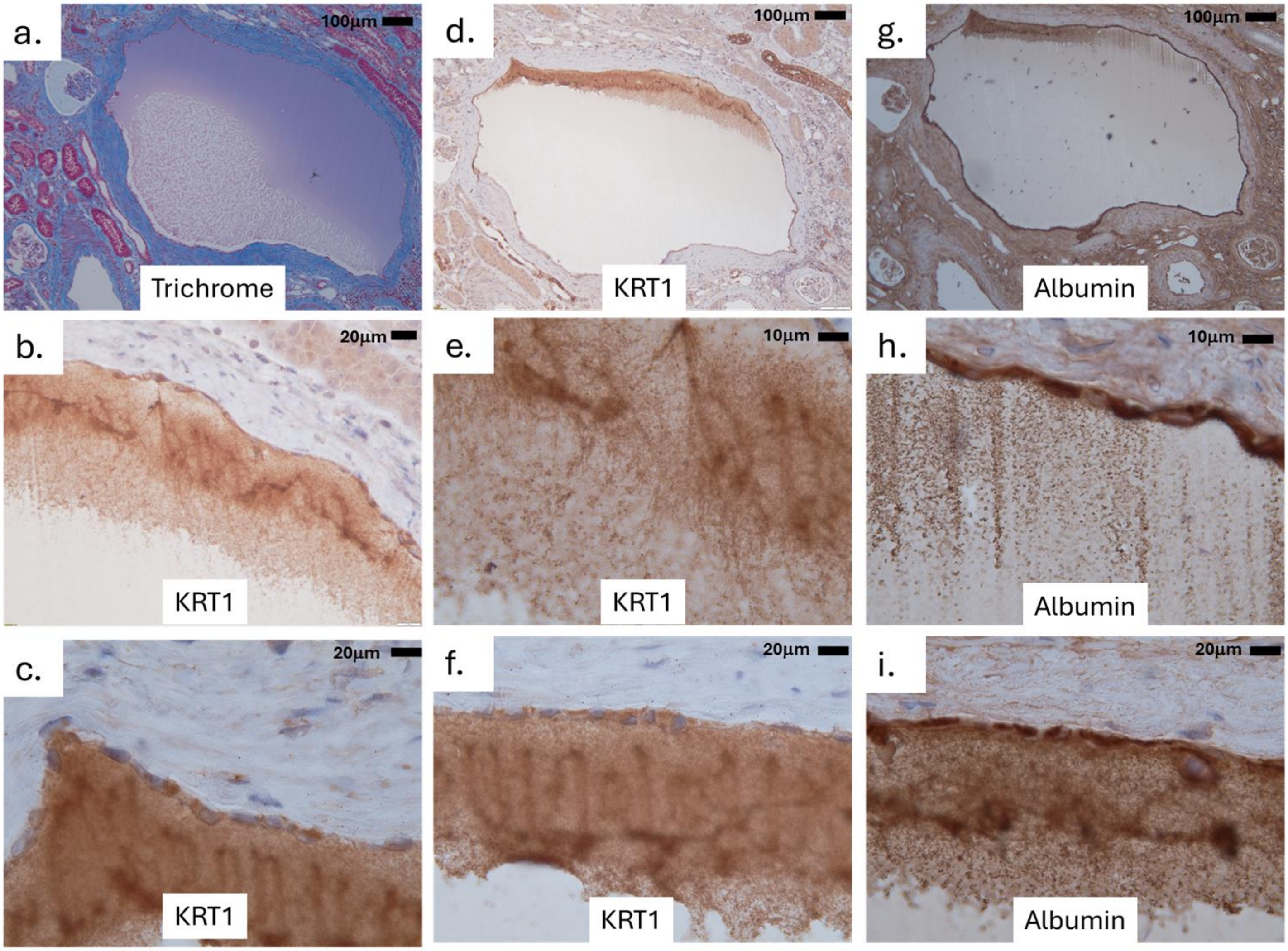


**Supplementary Figure 12**. **Albumin/Keratin staining of a large vascular cast from a human kidney.** One of twelve human kidneys examined was found to contain a large venous cast similar in appearance to that found in rats following ischemia. Like in rats this cast appeared blue with trichrome staining (**a**). The cast as well as the underlying venous endothelial cells were positive for Keratin-1 (**b-f**). The cast also stained positive for albumin (**g-i**).

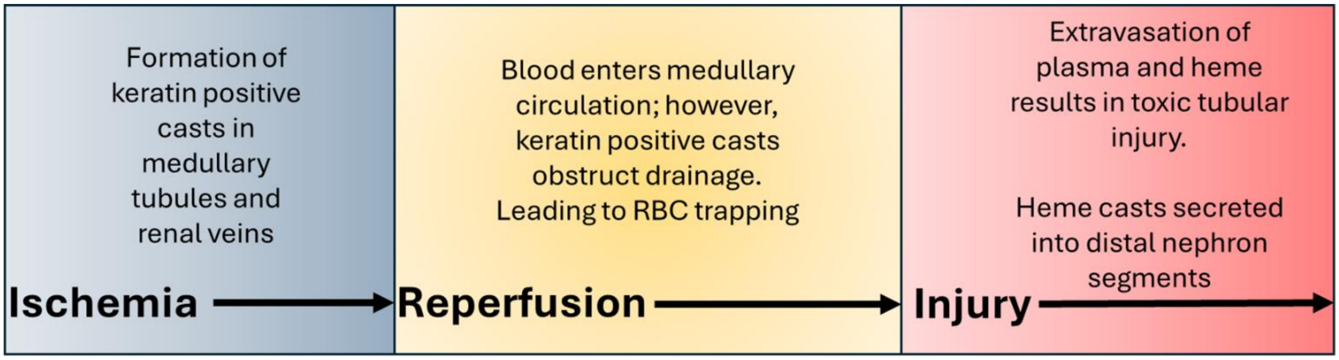


**Supplementary Figure 13**. **Summary figure outlining hypothesis**. Albumin/Keratin casts form during the ischemic period, obstructing tubules in the renal medulla as well as the medullary vessels and downstream renal veins. Upon reperfusion, blood first re-perfuses the renal medulla. Due to the obstruction in venous draining formed during ischemia, this blood cannot drain from the medulla so it accumulates in the outer-medullary capillary plexus. As blood continues to accumulate, the pressure inside the vasculature increases, forcing plasma to be extravasated into the medulla and cortical-medullary boundary zone. This extravasated plasma contains blood proteins and heme from damaged red blood cells within the congested vasculature which is toxic to the surrounding tubular epithelium. Heme casts are secreted by the tubular epithelium, eventually filling nephron segments downstream of the congested medulla.
